# Antagonistic effects of mitochondrial matrix and intermembrane space proteases on yeast aging

**DOI:** 10.1186/s12915-022-01352-w

**Published:** 2022-07-12

**Authors:** Montserrat Vega, David Castillo, Laura de Cubas, Yirong Wang, Ying Huang, Elena Hidalgo, Margarita Cabrera

**Affiliations:** 1 Oxidative Stress and Cell Cycle Group, Universitat Pompeu Fabra, C/ Dr. Aiguader 88, 08003 Barcelona, Spain; 2 BatchX Inc, San Jose, CA USA; 3 Jiangsu Key Laboratory for Microbes and Genomics, School of Life Sciences, Nanjing Normal University, 1 Wenyuan Road, Nanjing, 210023 China; 4 Department of Biology, Geology, Physics and Inorganic Chemistry, Rey Juan Carlos University, C/ Tulipán s/n, 28933 Móstoles, Madrid, Spain

**Keywords:** Chronological aging, Mitochondrial dynamics, Mitophagy, Mitoproteases, Respiratory capacity

## Abstract

**Background:**

In many organisms, aging is characterized by a loss of mitochondrial homeostasis. Multiple factors such as respiratory metabolism, mitochondrial fusion/fission, or mitophagy have been linked to cell longevity, but the exact impact of each one on the aging process is still unclear.

**Results:**

Using the deletion mutant collection of the fission yeast *Schizosaccharomyces pombe*, we have developed a genome-wide screening for mutants with altered chronological lifespan. We have identified four mutants associated with proteolysis at the mitochondria that exhibit opposite effects on longevity. The analysis of the respiratory activity of these mutants revealed a positive correlation between increased respiration rate and prolonged lifespan. We also found that the phenotype of the long-lived protease mutants could not be explained by impaired mitochondrial fusion/fission activities, but it was dependent on mitophagy induction. The anti-aging role of mitophagy was supported by the effect of a mutant defective in degradation of mitochondria, which shortened lifespan of the long-lived mutants.

**Conclusions:**

Our characterization of the mitochondrial protease mutants demonstrates that mitophagy sustains the lifespan extension of long-lived mutants displaying a higher respiration potential.

**Supplementary Information:**

The online version contains supplementary material available at 10.1186/s12915-022-01352-w.

## Background

With the growing elderly populations worldwide, increasing efforts have been focused on the study of aging-associated diseases and healthspan. Several biological processes have been linked with aging in different organisms. Among them, genomic instability, impaired proteostasis, and mitochondrial dysfunction constitute relevant hallmarks of aging [1].

In the last decades, numerous studies using yeast as a model system have contributed to our understanding of the aging process. Aging in yeast, like in many organisms, involves three basic features: reduced resistance to stresses, a decline in reproduction, and increased mortality. Moreover, calorie restriction, the most common anti-aging intervention, increases longevity in yeast and other model organisms including flies, nematodes, and mice [2]. The effect of calorie restriction is probably mediated by the inhibition of highly conserved nutrient-responsive kinases: TOR/SK6 and Ras/adenylate cyclase (AC)/PKA. Deletion of components of PKA and TOR pathways is known to induce lifespan extension in yeast through the activation of stress responses [3–5]. In the case of TOR inhibition, autophagy is stimulated and could also contribute to lifespan extension [6].

Two versions of lifespan exist in yeast: replicative and chronological lifespan [7]. Replicative lifespan (RLS) is defined by the number of daughter cells produced by a single cell before senescence. This process is equivalent to the longevity of proliferating cells in higher multicellular organisms. Chronological lifespan (CLS) represents the viability of a yeast cell during the stationary phase and recapitulates aging of non-proliferating cells. RLS and CLS are tightly connected and mutant strains with decreased replicative potential often exhibit a limited CLS.

Most research in yeast aging has been conducted using the budding yeast *Saccharomyces cerevisiae* (for reviews, see [8, 9]) but lately the fission yeast *Schizosaccharomyces pombe* has emerged as a powerful and complementary chronological aging model. Respiratory metabolism plays a central role in the regulation of chronological aging. In *S. pombe*, mutations in components of the electron transport chain (ETC) cause elevated reactive oxygen species (ROS) levels and shortening of CLS [10]. Protection from ROS is essential to ensure normal CLS as revealed by a double mutant lacking superoxide dismutase and with low glutathione levels [11]. This mutant is more sensitive to oxidative stress and displays increased levels of protein oxidation and shorter CLS.

To avoid the accumulation of oxidized and damaged proteins, mitochondria contain their own protein quality control (PQC) system, which includes two basic components: chaperones and proteases [12]. In both yeast models, chaperones of the Hsp90, Hsp70, and Hsp60 families and small Hsp (sHsp) assist in protein folding or refolding steps whereas ATP-dependent proteases are responsible for protein maturation or degradation. Mitochondria of fission yeast contain three main proteases: one soluble localized at the matrix called Lon1 and two membrane proteases, Yme1 which has its catalytic domains facing the intermembrane space and Yta12 with catalytic domains oriented towards the matrix. Moreover, the yeast protein Mgr3 serves as an adaptor for the protease Yme1 during the recognition of its substrates [13].

Controlled mitochondrial dynamics represents a second quality control pathway linked with the process of aging. In *S. cerevisiae*, mitochondrial fragmentation and reduced membrane potential have been reported with increasing replicative age, although it is unclear whether these events contribute to senescence [14, 15]. Notably, chronologically aged cells of fission yeast show reduced mitochondrial signal and loss of tubular mitochondria compared to young cells [16]. In budding yeast, loss of the mitochondrial fission machinery causes increased RLS whereas the absence of Mgm1 involved in fusion has been associated with shortening of RLS [17, 18]. Interestingly, a double mutant of *S. cerevisiae* with defective fusion and fission displays wild-type mitochondrial morphology, but decreased lifespan suggesting that the critical step in the control of lifespan is the balance between fusion and fission events and not the morphology [19].

A third strategy to ensure mitochondrial homeostasis is the removal of the organelle by mitophagy [20]. This process is initiated in the cytosol with the formation of a double-membrane structure (phagophore) that engulfs the damaged or superfluous mitochondria. After the sealing of the membrane around the organelle, the resulting autophagosome fuses with the lysosome (vacuole in yeast) where proteins and lipids are degraded. Most of the key components required in autophagy have been identified in budding yeast, including the receptor on the mitochondrial outer membrane (Atg32) recognized by the autophagy machinery [21]. Recently, two studies have also revealed the identity of specific components of the mitophagy pathway in fission yeast [22, 23].

Using the *S. pombe* deletion mutant collection, we have performed a genome-wide screening and identified four longevity mutants that are involved in mitochondrial protein quality control. While the absence of two proteases, Lon1 and Yta12, which reside in the matrix conferred a severe defect in CLS, mutant cells lacking the intermembrane space protease Yme1 and its adaptor protein Mgr3 were long-lived. The short-lived mutants displayed reduced oxygen consumption, low levels of mitochondrial DNA-encoded proteins, and impaired mitophagy, while the opposite characterized the long-lived mutants. Moreover, inhibition of mitophagy abolished the lifespan extension of both long-lived mutants implying that mitophagy is essential for delaying the aging process. On the other hand, we were not able to establish a direct connection between the longevity phenotypes and mitochondrial dynamics. In conclusion, the analysis of the mitochondrial function of these mutants reveals that increased respiratory potential and mitophagy act together to prolong lifespan by maintaining optimal mitochondrial function.

## Results

### Genome-wide screening for longevity deletion mutants in fission yeast

The classical method to monitor cell viability during chronological aging is based on the division potential of stationary phase cells when nutrients are replenished [7]. A possible caveat of this system is the identification of short-lived mutants that might be only affected in cell division, but not directly related to the aging process. To overcome this limitation, we have developed a genome-wide screening where cell viability is monitored by the uptake of a fluorescent dye (propidium iodide, PI) and flow cytometry [24]. Only dead cells with enhanced membrane permeability will be stained with PI.

**Fig. 1:**
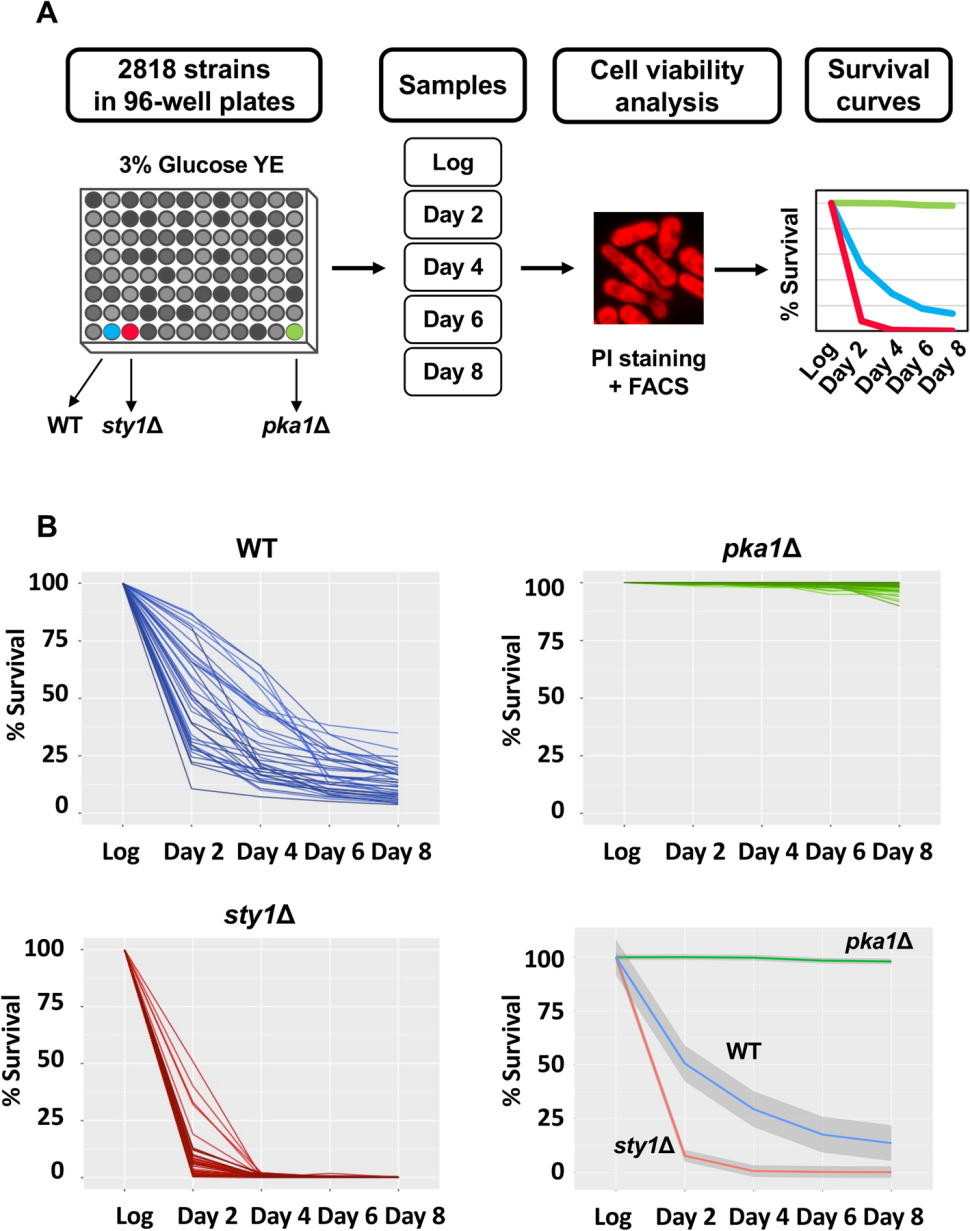
Chronological aging screening. **A** Experimental design for chronological aging screening. Bioneer collection plates were grown in rich media (YE) with 3% glucose, and samples were collected during logarithmic growth (Log) and days 2, 4, 6, and 8 after reaching the stationary phase. Cell viability was measured by FACS using cell staining with propidium iodide (PI). **B** Survival curves of control strains. Percentage of survival of 666 (WT), *sty1*Δ (short-lived), and *pka1*Δ (long-lived) strains at the indicated time points. Shadows in average curves indicate a 95% confidence interval

A total of 2818 deletion mutants (Bioneer collection version 2) were grown in 96-well plates in rich media containing 3% glucose (Fig. 1A, Additional file 1: Table S1). Each plate also included three control strains: wild type (WT), *sty1*Δ (short-lived), and *pka1*Δ (long-lived) [5, 25]. Both kinases, Sty1 and Pka1, are well-characterized by their function in stress response and nutrient signaling, respectively. Every 2 days until day 8, cells from aged cultures were stained with PI and analyzed by flow cytometry to quantify the non-stained and viable population. The fraction of viable cells at each time point allowed us to represent the survival curve of each deletion mutant. Figure 1B shows the survival curves of wild type, *sty1*Δ, and *pka1*Δ from different plates or experiments and the average curve for each control strain, where short- or long-lived mutants can be clearly distinguished from wild-type cells.

### Around 500 deletion mutants display altered longevity in rich media

**Fig. 2:**
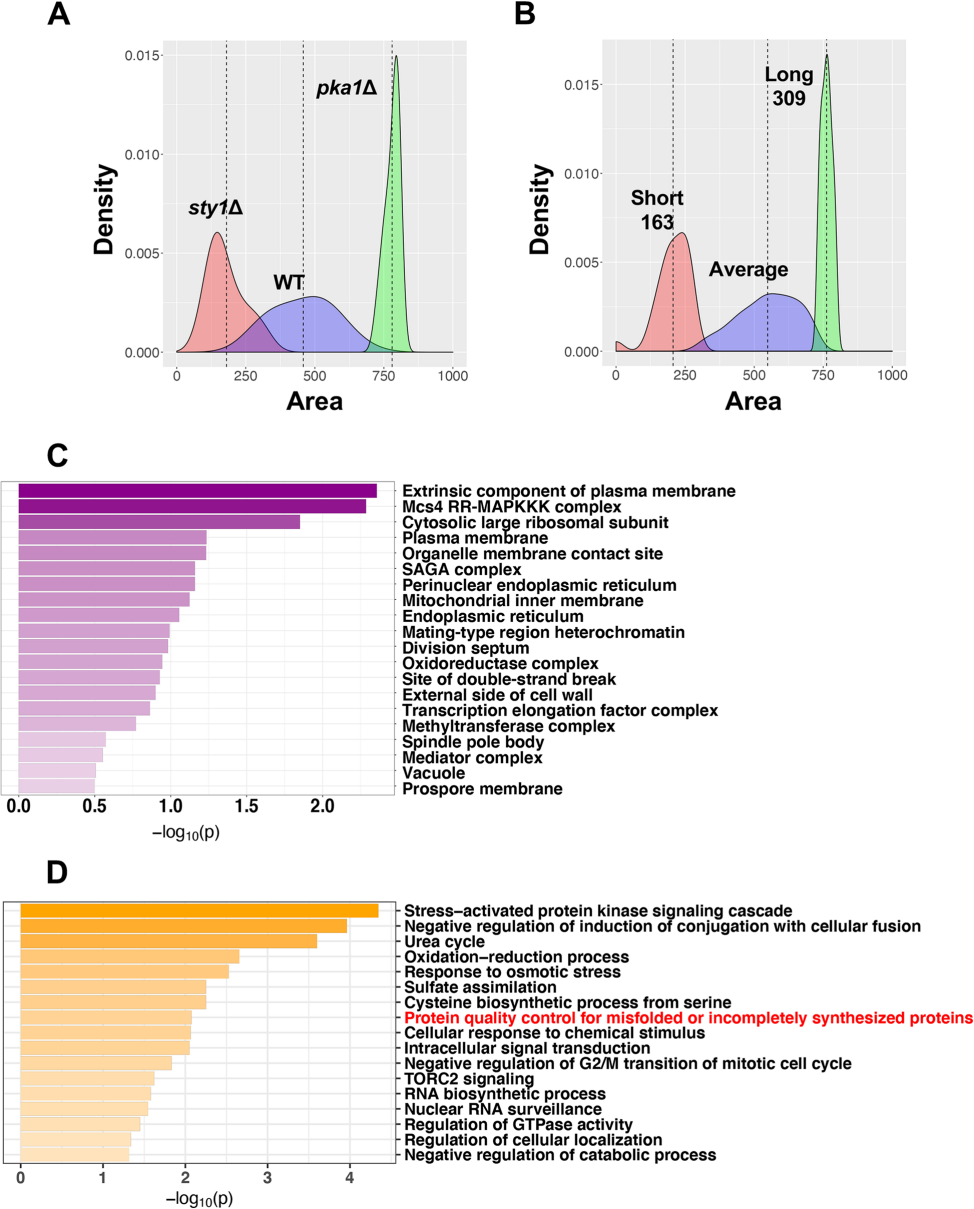
Classification of chronological aging mutants and Gene Ontology (GO) enrichment analysis. **A**, **B** Area distribution of the control strains used in the screening. Density plot showing the survival distribution of the three control strains: 666 (WT), *sty1*Δ (short-lived strain), and *pka1*Δ (long-lived strain). These distributions were considered to estimate the probability for every analyzed mutant to belong to each one of these groups. Once these posterior probabilities were calculated, each mutant was assigned to the category with the highest probability. The density plot (**B**) shows the resulting classification of mutants based on this approach. **C**, **D** Gene Ontology term enrichment. The long-lived (309) and short-lived (163) mutants identified in the screening were selected for GO enrichment analysis. They were classified into Cellular Component (**C**) and Biological Process (**D**) GO terms using Metascape [64]. Each bar represents a GO term ordered by statistical significance (-log10 *p *value)

We chose the area under the survival curve as the ideal parameter to compare the longevity of the mutants and control strains (wild type, *sty1*Δ, and *pka1*Δ). We first obtained the area distribution of the control strains and used a Bayesian approximation to estimate the probability of each mutant to belong to the different distributions (Fig. 2A). The survival of most mutants reproduced the wild-type distribution, 163 were classified as short-lived mutants, and 309 were identified as long-lived mutants (Fig. 2B, Additional file 2: Table S2). The larger number of long-lived mutants could be explained by the growth in rich media, which favors the identification of those mutants that mimic lifespan extension by calorie restriction [5].

To highlight the biological processes with more impact on CLS, we performed a Gene Ontology (GO) enrichment analysis including all mutants with altered longevity. We identified a few enriched cellular components, which are involved in plasma membrane organization, oxidative stress response, and ribosome function (Fig. 2C, Additional file 3: Table S3). As expected, several biological processes that have been linked previously to aging, such as stress response, cell cycle progression, and protein quality control (PQC) were overrepresented (Fig. 2D, Additional file 3: Table S3). This last term (PQC for misfolded or incompletely synthesized proteins) caught our attention since it includes two genes coding for Lon1 and Mgr3, both involved in mitochondrial protein turnover, but with opposite longevity phenotypes. Lack of the mitochondrial protease Lon1 reduced longevity whereas the absence of Mgr3, adaptor protein of the protease Yme1 [13], led to increased lifespan. We chose to deepen into the analysis of these mutants, since their opposite phenotypes highlight the complexity of the effects of mitochondrial homeostasis in cell longevity.

### Cells lacking mitochondrial proteases are affected in their longevity profiles

**Fig. 3:**
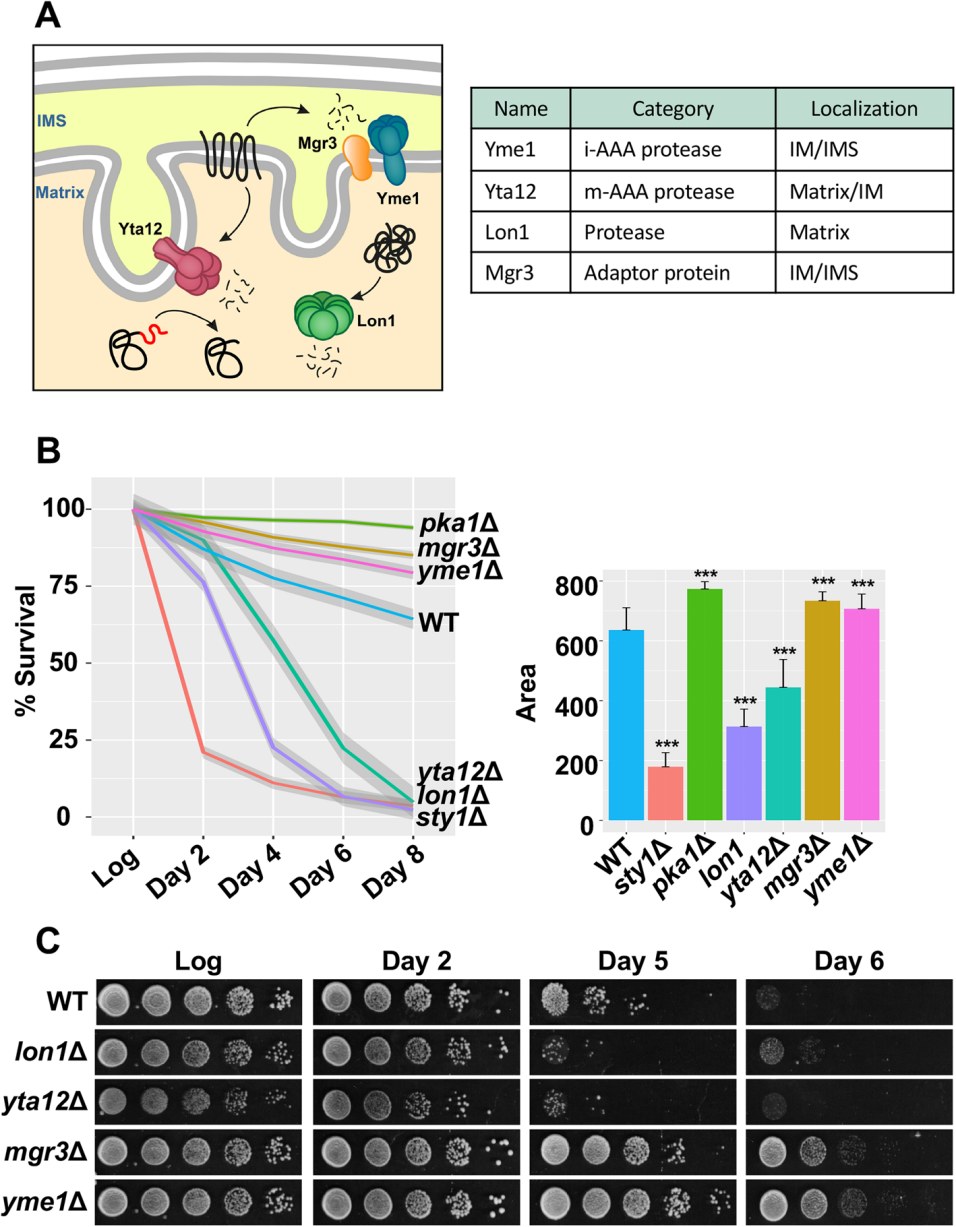
Deletion of mitochondrial proteases affects longevity. **A** Scheme and table depicting the localization of the mitochondrial proteases Lon1, Yta12, Yme1, and the adaptor protein Mgr3. **B**, **C** Deletion of the mitoproteases Lon1 or Yta12 decreases lifespan, whereas deletion of Mgr3 or Yme1 increases longevity. Strains 972 (WT), *lon1*Δ, *yta12*Δ, *mgr3*Δ, and *yme1*Δ were grown in rich media with 3% glucose and viability was measured by FACS using propidium iodide (**B**) and survival spots (**C**). **B** Line plot represents the local regression curves for the average survival of each strain (*n* > 30) at different time points. Each survival curve also displays a 95% confidence interval band. Bar plot depicts the average area under the survival curve of each strain, and error bars represent SD. Significant differences between deletion strains and wild type were determined by two-sided *t*-test (*p < 0.05, **p < 0.01, ***p < 0.001). **C** Serial dilutions of culture samples from the logarithmic phase (Log) and days 2, 5, and 6 of the stationary phase were spotted onto rich media plates

To analyze the role of mitochondrial PQC in chronological aging, we decided to examine the longevity of another two mitoprotease mutants, *yme1*Δ and *yta12*Δ, which were missing in our deletion collection. Both proteases form multimeric complexes localized in the inner mitochondrial membrane and contain conserved AAA + domains for membrane extraction of their substrates. Yme1 and Yta12 are known as i-AAA and m-AAA proteases, respectively, because their catalytic domains are exposed to opposite sides of the inner mitochondrial membrane (Fig. 3A). As mentioned above, mitochondria in *S. pombe* hold a third ATP-dependent protease Lon1, which is localized in the matrix, and the adaptor protein Mgr3 acting together with Yme1 in the proteolysis of mitochondrial membrane proteins (Fig. 3A).

Using the flow cytometry-based assay and prototrophic strains, we recapitulated the opposite longevity phenotypes of *lon1*Δ and *mgr3*Δ mutants (Fig. 3B). Moreover, cells lacking the protease Yme1 displayed increased lifespan in a similar manner to *mgr3*Δ mutant, whereas the loss of the protease Yta12 resulted in a significant reduction of longevity (Fig. 3B). Next, we analyzed the viability of these four deletion mutants monitoring how cells resume growth after chronological aging. We performed the traditional assay, where aged cells from different time points (days 2–6) were spotted in rich media to allow growth (Fig. 3C). This alternative method confirmed the longevity phenotypes of the four mutants; *lon1*Δ and *yta12*Δ were classified as short-lived mutants whereas *mgr3*Δ and *yme1*Δ cells exhibited increased survival under chronological aging (Fig. 3C).

### The short- or long-lived protease mutants display decreased or enhanced respiratory rates, respectively

**Fig. 4:**
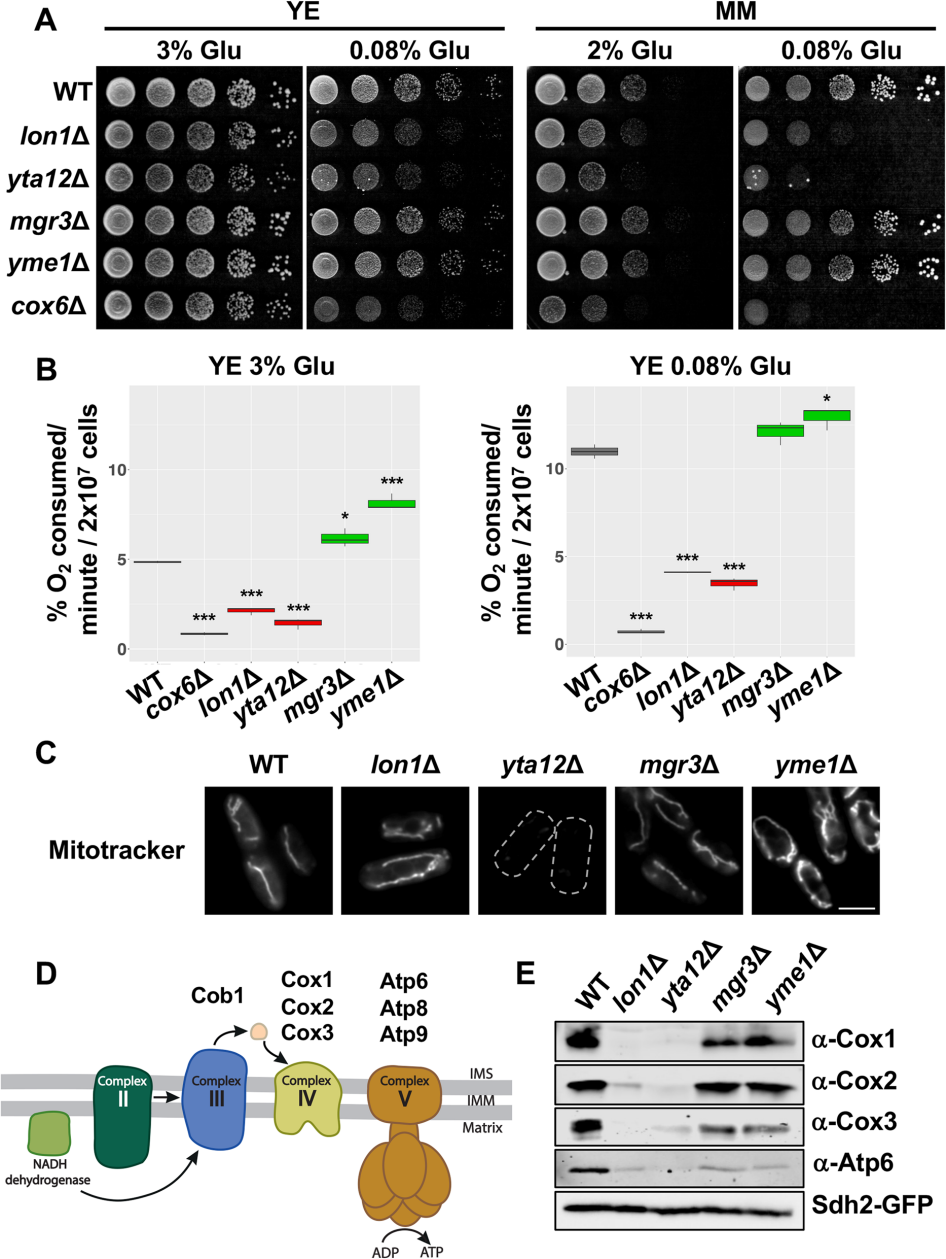
Short-lived strains, *lon1*Δ and *yta12*Δ, display dysfunctional mitochondria. **A** *lon1*Δ and *yta12*Δ exhibit a growth defect under low glucose conditions. 972 (WT), *lon1*Δ, *yta12*Δ, *mgr3*Δ, *yme1*Δ, and *cox6*Δ strains were grown in rich media (YE) with 3% glucose and minimal media (MM) with 2% glucose. Serial dilutions of logarithmically growing cells were spotted onto YE containing 3% or 0.08% glucose and MM containing 2% or 0.08% glucose plates. **B** Differences in oxygen consumption levels of mitoprotease mutants and *mgr3*Δ. The indicated strains were grown in rich media with 3% or 0.08% glucose. Oxygen consumption was measured when cultures reached an OD_600_ of 0.5. Data from three biological replicates are shown. Significant differences between deletion strains and wild type were determined by two-sided *t*-test (*p < 0.05, **p < 0.01, ***p < 0.001). **C** Mitochondrial membrane potential (ΔΨ) is reduced in cells lacking the protease Yta12. Mitochondria of the indicated strains were stained with MitoTracker red and ΔΨ was determined by fluorescence microscopy. Maximum and minimum levels were adjusted using Fiji (ImageJ, National Institutes of Health) [65]. The corresponding quantification is shown in Fig. S1. Scale bar, 5 μm. **D** Scheme depicting the localization of OXPHOS proteins encoded by mitochondrial DNA (mtDNA). **E** Short-lived strains have diminished steady-state levels of several mtDNA-encoded proteins. Mitochondrial TCA extracts of 972 (WT), *lon1*Δ, *yta12*Δ, *mgr3*Δ, and *yme1*Δ were analyzed by immunoblotting using antibodies against the mtDNA-encoded proteins Cox1, Cox2, Cox3, and Atp6. Sdh2-GFP was used as a loading control

To better understand the possible functions of the mitochondrial proteases in aging, we focused on the analysis of the respiratory activity of the deletion mutants. We first examined the growth of the mutants in rich or minimal media with low glucose concentration (0.08% Glu) that promote respiratory metabolism (Fig. 4A). We observed that the short-lived mutants *lon1*Δ and *yta12*Δ showed a severe growth defect in respiratory-prone media, comparable to that detected in *cox6*Δ lacking a subunit of the ETC complex IV [5]. In contrast, long-lived mutants *mgr3*Δ and *yme1*Δ displayed a normal growth in low glucose conditions (Fig. 4A). In concordance with these results, when we monitored oxygen consumption of the mutants, cells lacking Lon1 or Yta12 displayed decreased respiratory capacity in high and low glucose conditions whereas the loss of Mgr3 or Yme1 was associated with enhanced respiration (Fig. 4B). This increase in the respiratory activity was more evident for *yme1*Δ mutant in high glucose medium (Fig. 4B, left panel).

Another relevant parameter related to respiratory metabolism is the maintenance of the mitochondrial membrane potential (ΔΨ), which in turn is required for protein import into the mitochondria [26]. Using MitoTracker, a ΔΨ-dependent fluorescent dye, we detected a significant loss of ΔΨ in *yta12*Δ mutant and increased ΔΨ in *yme1*Δ cells, both results in consonance with our previous findings (Fig. 4C, Additional file 4: Fig. S1).

We presumed that the respiratory deficiency caused by the lack of the proteases Lon1 or Yta12 could be a consequence of impaired synthesis or assembly of the oxidative phosphorylation (OXPHOS) components encoded by the mitochondrial genome. To confirm this possibility, we isolated mitochondria from wild-type and deletion mutants and monitored the levels of several mitochondrial DNA (mtDNA)-encoded OXPHOS proteins by western blot. In *S. pombe*, seven subunits of the OXPHOS complexes are encoded by mtDNA, one belongs to complex III, three to complex IV, and three are part of the ATP synthase or complex V (Fig. 4D). Immunoblots in Fig. 4E show that the levels of Cox1, Cox2, Cox3, and Atp6 proteins are similar in wild-type and the long-lived mutants *mgr3*Δ and *yme1*Δ. However, the blots demonstrate highly reduced levels of these proteins in *lon1*Δ and *yta12*Δ mutants which might explain the respiratory defects observed in these strains.

To uncover the consequences of these alterations of the OXPHOS system, we measured H_2_O_2_ abundance at the mitochondrial matrix by expressing the peroxide reporter HyPer7 at this subcellular compartment and determining its oxidation levels [27, 28]. The percentage of oxidation of this reporter depends on the steady-state H_2_O_2_ levels at the matrix, being around 40% (OxD_0_ of 0.4) in wild-type cells and increasing up to 60% when the main H_2_O_2_ scavenger, Tpx1, is missing [28]. As shown in Fig. S2, the degree of oxidation of the probe in the long-lived mutants *mgr3*Δ and *yme1*Δ was undistinguishable from wild-type cells. In contrast, the *yta12*Δ strain displayed enhanced levels of basal probe oxidation (OxD_0_ of 0.46) at the mitochondrial matrix compared to the wild-type strain (OxD_0_ of 0.4), indicative of higher steady-state levels of H_2_O_2_ (Additional file 5: Fig. S2). Cells lacking Lon1 displayed a slight increase in basal oxidation of MTS-Hyper7, suggesting that both short-lived mutants show enhanced production of mitochondrial ROS (Additional file 5: Fig. S2).

In summary, low respiratory capacity and enhanced ROS production correlate with a decrease in chronological lifespan whereas enhanced respiration might promote longevity.

### Mitochondrial dynamics as an important process modulating aging

**Fig. 5:**
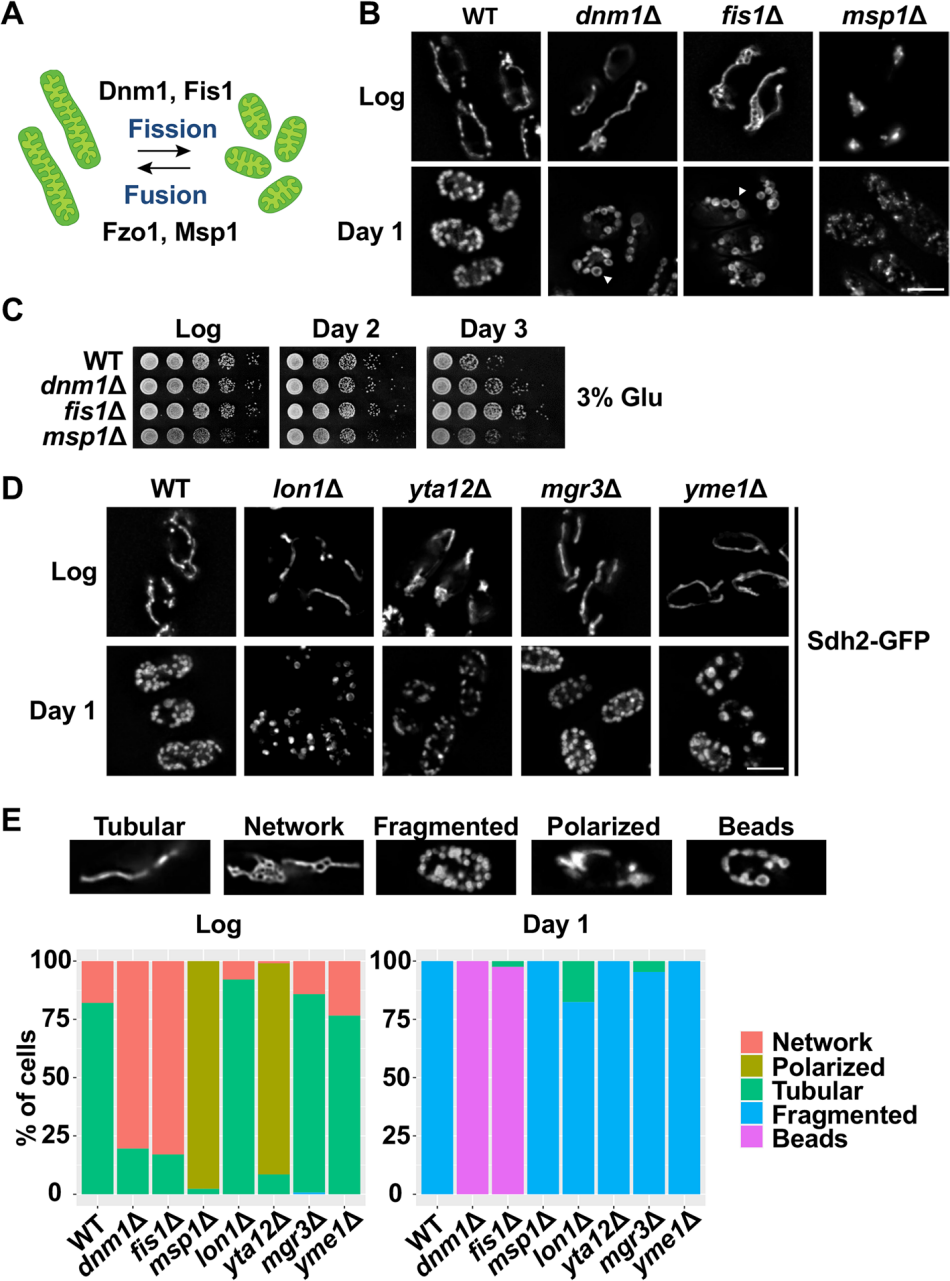
Influence of mitochondrial dynamics on chronological aging. **A** Scheme depicting the different proteins involved in mitochondrial fusion and fission. Dnm1 and Fis1 proteins mediate mitochondrial fission while Fzo1 and Msp1 are dedicated to mitochondrial fusion. **B** Mitochondrial morphology changes during chronological aging were determined by fluorescence microscopy in 972 (WT), *dnm1*Δ, *fis1*Δ, and *msp1*Δ strains expressing Sdh2-GFP. Cells were grown in rich media containing 3% glucose and analyzed after logarithmic growth (Log) and day 1 of the stationary phase. White arrows indicate mitochondria arranged like beads on a string. Scale bar, 5 μm. **C** *dnm1*Δ and *fis1*Δ strains show enhanced longevity. 972 (WT), *dnm1*Δ, *fis1*Δ, and *msp1*Δ cells were grown in rich media containing 3% glucose. Serial dilutions corresponding to culture samples from the logarithmic phase (Log) and days 2 and 3 of the stationary phase were spotted onto rich media plates. **D** Analysis of mitochondrial morphology in mitoprotease mutants and *mgr3*Δ. Fluorescence microscopy images of 972 (WT), *lon1*Δ, *yta12*Δ, *mgr3*Δ, and *yme1*Δ strains expressing the mitochondrial marker Sdh2-GFP. Cells were grown in rich media containing 3% glucose and analyzed after logarithmic growth (Log) and day 1 of the stationary phase. Scale bar, 5 μm. **E** Classification of mitochondrial morphology phenotypes. Mitochondria from 972 (WT), *dnm1*Δ, *fis1*Δ, *msp1*Δ, *lon1*Δ, *yta12*Δ, *mgr3*Δ, and *yme1*Δ were manually classified into five categories: tubular, network, fragmented, polarized, and beads. Images represent an example of each category. Bar plots show the percentage of cells from each category during logarithmic growth (Log) and day 1 of the stationary phase (*n* > 100)

Membrane fusion and fission are essential processes to preserve the distinctive morphology and number of healthy mitochondria. In fission yeast, mitochondrial fragmentation or fission is mediated by a dynamin-related GTPase Dnm1, which is recruited to the outer membrane (OM) by its receptor Fis1. Dnm1 forms a ring structure on the OM promoting membrane constriction and fission. Mitochondrial fusion instead is driven by the interaction of dynamin-like GTPases from opposing membranes [29]. In *S. pombe*, Fzo1 and Msp1 present in the OM and IM, respectively, are responsible for mitochondrial fusion (Fig. 5A).

To establish an association between mitochondrial dynamics and longevity, we first analyzed possible changes in mitochondrial morphology during chronological aging. We observed a severe mitochondrial fragmentation in wild-type aged cells grown in high glucose media (Fig. 5B). Additionally, fragmented mitochondria were accompanied by a reduction of ΔΨ measured using MitoTracker fluorescence (Additional file 6: Fig. S3). Similar events have been reported in budding yeast during replicative aging [14]. By contrast, mitochondrial fragmentation was only observed in aged cells of the fusion mutant *msp1*Δ and not in *dnm1*Δ and *fis1*Δ strains (Fig. 5B). In these two mutants, mitochondria were arranged like beads on a string (Fig. 5B), structures that may result from defective cristae formation [30].

Studies in model organisms have shown conflicting results about the impact of inhibiting mitochondrial fission on aging [17, 31]. Thus, both deletion and overexpression of the fission protein dynamin have been reported to increase lifespan. Monitoring cell survival during chronological aging, we have found that deletion of the fission genes dnm1 or fis1 caused a significant increase of longevity whereas the loss of the fusion GTPase Msp1 had no effect on lifespan (Fig. 5C). This result is consistent with the increased RLS observed upon deletion of Dnm1 or Fis1 in budding yeast [17].

Considering these results, we hypothesized whether the longevity phenotype of the mitoprotease mutants could be explained by any alteration in their fusion-fission equilibrium. We used an image analysis tool (MiNA) [32] to measure the length and branches of mitochondrial structures in logarithmically growing cells. While the fusion and fission mutants displayed decreased and enhanced elongation capacities, respectively, the protease mutants *lon1*Δ, *mgr3*Δ, and *yme1*Δ did not differ from the control, dismissing a straightforward correlation between longevity and mitochondrial fusion/fission (Fig. 5D, Additional file 7: S4). To better characterize possible changes in mitochondrial morphology triggered by aging, we sorted the observed structures into five classes: network, polarized, tubular, fragmented, and beads (Fig. 5E). After logarithmic growth, wild-type, *lon1*Δ, *mgr3*Δ, and *yme1*Δ strains showed tubular mitochondria, whereas mitochondria in *dnm1*Δ and *fis1*Δ mutants were distributed as a branched network (Fig. 5D, E). Interestingly, the short-lived mutant *yta12*Δ displayed aggregated mitochondria at the cell poles similar to *msp1*Δ cells, suggesting that this matrix protease may participate in the regulation of mitochondrial fusion (Fig. 5D, E). Upon chronological aging, all mitochondrial protease mutants exhibited fragmented mitochondria as described earlier for wild-type cells (Fig. 5D, E). Thus, we conclude that the longevity phenotypes of our mitochondrial protease mutants cannot be explained by their mitochondrial fusion and fission properties.

### During chronological aging, mitophagy contributes to lifespan extension

**Fig. 6:**
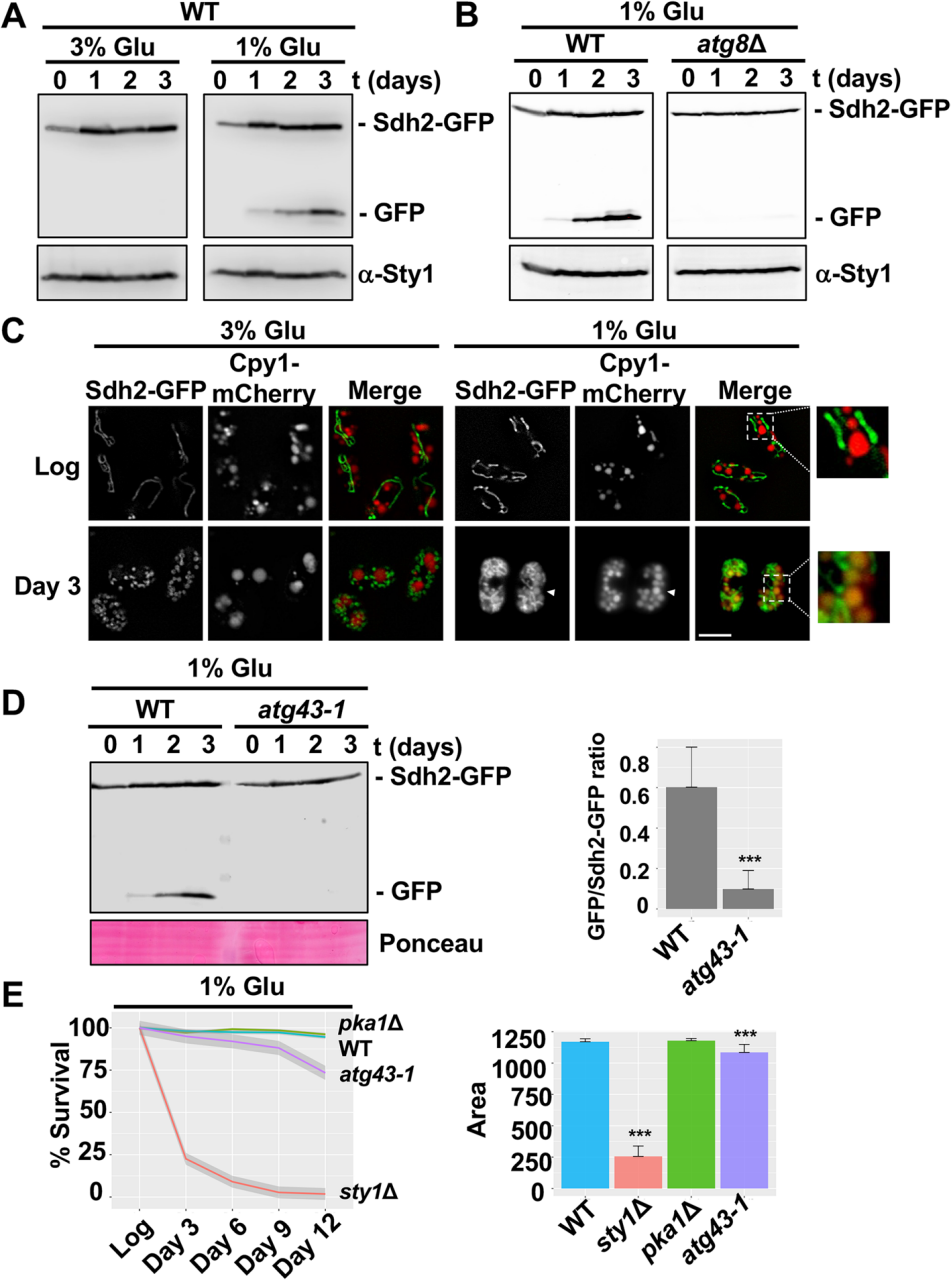
Impact of mitophagy inhibition on chronological lifespan. **A** Mitophagy is induced during the stationary phase in low glucose conditions. Mitophagy was monitored by the cleavage of Sdh2-GFP detected by immunoblotting with an anti-GFP antibody. A wild-type strain (972) was grown in rich media containing 3% glucose (3% Glu) or 1% glucose (1% Glu). Samples correspond to TCA extracts of cell cultures after logarithmic growth (day 0) and days 1, 2, and 3 of the stationary state. Sty1 levels were used as a loading control. **B** Deletion of Atg8 inhibits processing of Sdh2-GFP during the stationary phase. Mitophagy induction in 972 (WT) and *atg8*Δ strains was monitored by immunoblotting as in **A**. Samples correspond to TCA extracts from logarithmic growth (day 0) and days 1, 2, and 3 of the stationary state. Sty1 levels were used as a loading control. **C** Colocalization of the mitochondrial marker Sdh2-GFP and the vacuolar marker Cpy1-mCherry. Wild-type cells grown in rich media with 3% glucose (3% Glu) or 1% glucose (1% Glu) were visualized during logarithmic growth (Log) and day 3 of the stationary phase. Insets show a magnified region containing vacuoles labeled with GFP and Cpy1-mCherry. Scale bar, 5 μm. **D** *atg43-1* mutant blocks mitophagy during the stationary phase at low glucose conditions. Mitophagy in 972 (WT) and *atg43-1* strains was monitored by the cleavage of Sdh2-GFP detected by immunoblotting with an anti-GFP antibody. Samples correspond to TCA extracts of cell cultures grown in media with 1% glucose and collected after logarithmic growth (day 0) and days 1, 2, and 3 of the stationary state. Ponceau was used as the loading control. Bar plot represents the mean and SD of the GFP/Sdh2-GFP ratio from three independent experiments. Quantification was performed using Image Lab software. Significant differences between the *atg43-1* strain and wild type were determined by a two-sided *t*-test (***p < 0.001). **E** Inhibition of mitophagy reduces cell longevity under low glucose conditions (1% Glu). Lifespan of 972 (WT), *pka1*Δ, *sty1*Δ, and *atg43-1* strains was measured by propidium iodide staining and FACS. Line plot represents the local regression curves for the average survival of each strain (*n* > 10) at different time points. Each survival curve also displays a 95% confidence interval band. Bar plot depicts the average area under the curve of each strain, and error bars represent SD. Significant differences between deletion strains and wild type were determined by a two-sided *t*-test (*p < 0.05, **p < 0.01, ***p < 0.001)

Several studies demonstrate the anti-aging role of mitophagy facilitating the removal of dysfunctional or damaged mitochondria [33–35]. To uncover the relevance of mitophagy during chronological aging, we first analyzed mitophagy induction in cells grown for different time points in rich (3% Glu) and low glucose (1% Glu) media (Fig. 6A). Mitophagy was monitored here by the cleavage of the mitochondrial protein Sdh2-GFP and the release of GFP which occurs during mitochondria degradation by autophagy [21]. Using this assay, we only detected mitophagy activation in low glucose conditions that promote respiratory growth and hence the turnover of mitochondria (Fig. 6A). Moreover, the degradation of Sdh2-GFP observed under low glucose conditions was inhibited in the autophagy mutant *atg8*Δ, demonstrating unequivocally that this process is autophagy-dependent (Fig. 6B). In agreement with these results, Sdh2-GFP only colocalized with a vacuole marker (Cpy1-mCherry) in aged cells after growth in a low glucose medium (Fig. 6C).

Recently, the mitophagy receptor Atg43 has been identified in fission yeast allowing the study of the specific role of mitophagy in lifespan extension [23]. To this end, we examined first whether Atg43 is required for mitophagy during chronological aging. Using low glucose conditions and *atg43-1* mutant with a partial deletion of the gene, we observed a complete inhibition of the mitophagy process (Fig. 6D), resembling the effect reported under nitrogen starvation [23]. In the next step, we assayed the lifespan of the *atg43-1* mutant in low glucose conditions and found a decrease in longevity compared to the wild-type strain (Fig. 6E, Additional file 8: S5A), supporting the need of active mitophagy to prevent premature aging. Similar results were obtained in budding yeast using the mutant lacking the mitophagy receptor Atg32, but under severe caloric restriction conditions [35].

In *S. pombe*, three Atg proteins, Atg20, Atg24, and Atg24b, have also been implicated in the degradation of mitochondria by autophagy [22]. Comparable to the *atg43-1* mutant (Fig. 6D, E), double deletion of these Atg proteins led to impaired mitophagy (Additional file 8: Fig. S5B) and decreased survival (Additional file 8. Fig. S5C) during chronological aging after growth in low glucose media.

**Fig. 7:**
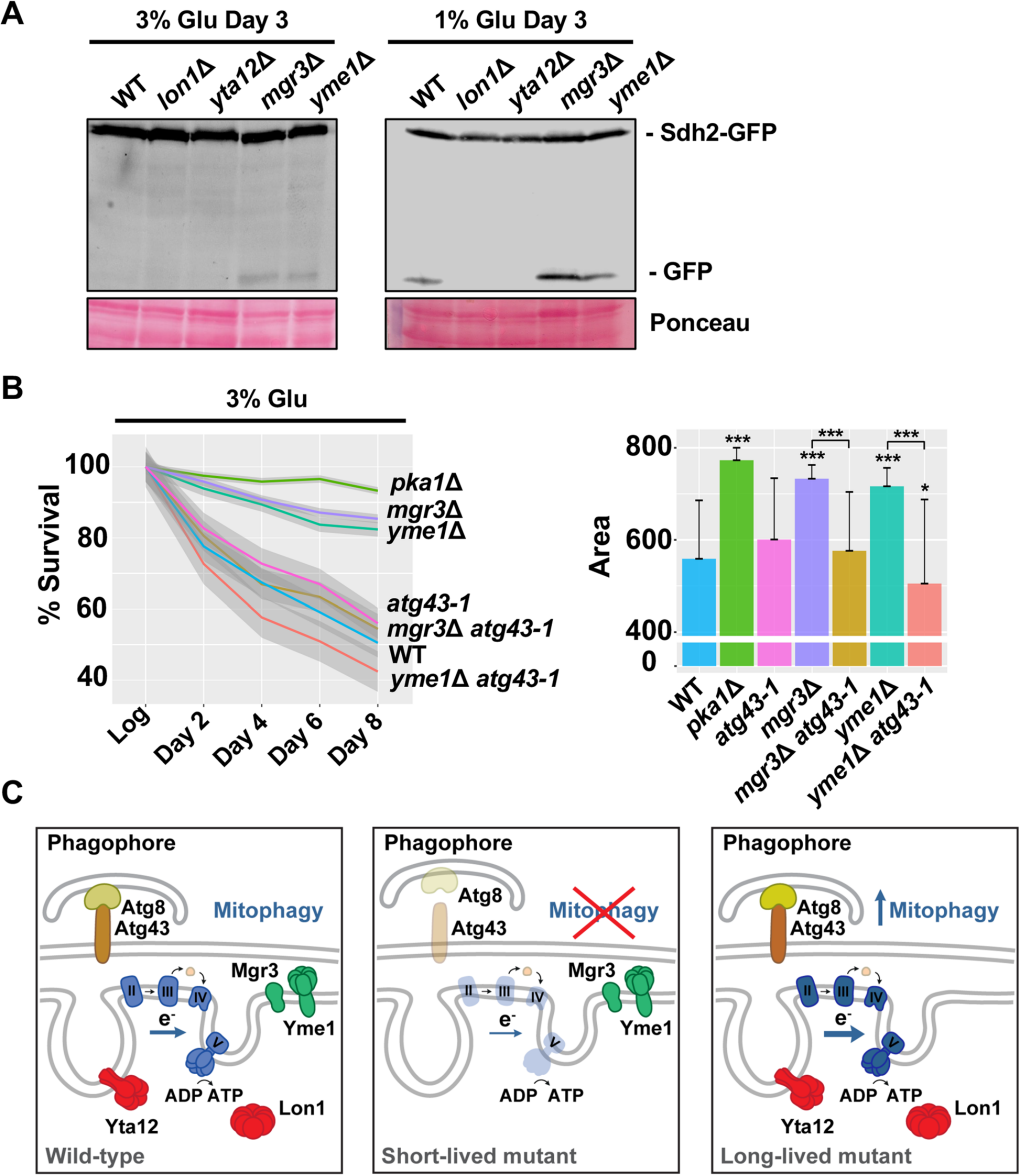
Mitophagy is required for lifespan extension in the long-lived mutants *mgr3*Δ and *yme1*Δ. **A** Monitoring mitophagy induction in *lon1*Δ, *yta12*Δ*, mgr3*Δ, and *yme1*Δ strains*.* The experiment was performed as in Fig. 6A. Samples correspond to cells grown in rich media containing 3% or 1% glucose and day 3 of the stationary phase. Ponceau was used as the loading control. Quantification of mitophagy activation is shown in Fig. S6A. **B** Mitophagy contributes to the longevity extension observed in *mgr3*Δ and *yme1*Δ strains under high glucose conditions (3% Glu). Lifespan of 972 (WT), *pka1*Δ, *mgr3*Δ, *yme1*Δ, *atg43-1*, *mgr3*Δ *atg43-1*, and *yme1*Δ *atg43-1* strains was measured by propidium iodide staining and FACS. Line plot represents the local regression curves for the average survival of each strain (*n* > 10) at different time points. Each survival curve also displays a 95% confidence interval band. Bar plot depicts the average area under the curve of each strain, and error bars represent SD. Significant differences between deletion strains and wild type, and *mgr3*Δ and *yme1*Δ mutants versus *mgr3*Δ *atg43-1* and *yme1*Δ *atg43-1*, respectively, were determined by a two-sided *t*-test (*p < 0.05, **p < 0.01, ***p < 0.001). **C** Model depicting the effect of deletion of the three mitochondrial proteases and the adaptor protein Mgr3 on longevity. Short-lived mutants, *lon1*Δ and *yta12*Δ, are characterized by impaired respiration and loss of mitophagy which curtail cell survival during the stationary phase. Conversely, in long-lived mutants, *mgr3*Δ and *yme1*Δ, enhanced respiratory activity together with the elimination of damaged mitochondria by mitophagy contribute to the extension of chronological lifespan

We questioned whether mitophagy activation could mediate lifespan extension in the long-lived mutants *mgr3*Δ and *yme1*Δ. To test this possibility, we analyzed Sdh2-GFP processing in the mitoprotease deletion mutants and *mgr3*Δ after growth in high and low glucose media. *mgr3*Δ and *yme1*Δ mutants with increased longevity displayed enhanced mitophagy induction compared to wild-type strain, specially *mgr3*Δ cells, where mitophagy was stimulated even in rich media (Fig. 7A, Additional file 9: Fig. S6A). By contrast, mitophagy induction during chronological aging was not detected in the short-lived strains *lon1*Δ and *yta12*Δ (Fig. 7A, Additional file 9: Fig. S6A). These results point to a potential association between mitophagy activation and increased lifespan.

To confirm if mitophagy contributes to the increase in longevity observed in *mgr3*Δ and *yme1*Δ, we analyzed the CLS of the double mutants grown in high glucose media. Defective Atg43 and in turn impaired degradation of mitochondria reduced the longevity of both mutants to wild-type levels indicating that mitophagy induction is critical for the lifespan extension of *mgr3*Δ and *yme1*Δ cells (Fig. 7B). Interestingly, *atg43-1* did not reduce the lifespan in a wild-type background and high glucose media (Fig. 7B), supporting the idea that mitophagy is required to sustain improved longevity in a context of increased respiration, such as wild-type cells under calorie restriction (Fig. 6E) [35] and long-lived deletion mutants under glucose-rich conditions (Fig. 7B). In contrast, mitophagy inhibition by *atg43-1* had no negative effect on the lifespan of the short-lived mutants *lon1*Δ and *yta12*Δ (Additional file 9: Fig. S6B). Considering our findings, we can conclude that functional mitophagy represents a common anti-aging strategy shared by worms, flies, and yeast models.

## Discussion

Using a genome-wide screening, we have identified almost 500 deletion mutants of fission yeast with altered lifespan, among them four mutants associated with protein maturation/degradation at the mitochondria. The analysis of different mitochondrial processes in these mutants revealed that increased respiration can mediate a significant extension of chronological lifespan when it is combined with enhanced mitophagy (Fig. 7B, C). Both processes guarantee cell survival during chronological aging; a high respiratory activity ensures an efficient energy production whereas mitophagy is required to maintain a functional pool of mitochondria. Our results also support that the positive effect of respiration on longevity is achieved during logarithmic growth; during this period of adaptation, cells become prepared to survive in more adverse conditions. On the other hand, low respiratory metabolism caused by OXPHOS-deficient mitochondria, and mitophagy inhibition are associated with premature chronological aging. Moreover, we uncovered possible connections of the mitochondrial PQC, and in particular the activity of some mitoproteases and the adaptor protein Mgr3 with the synthesis of mtDNA-encoded ETC components, the induction of mitophagy, and the regulation of mitochondrial fusion/fission events.

In our screening, *lon1*Δ and *yta12*Δ have been characterized as short-lived mutants. Both deletion strains showed poor growth in respiratory media and reduced oxygen consumption (Fig. 4A, B). This respiratory deficiency could be explained at least partially by a decrease in the levels of OXPHOS components (Fig. 4E). Studies in budding yeast have previously linked deletion of OXPHOS subunits or inhibition of complex III with reduced respiration and decreased CLS [36, 37]. Likewise, deletion of ETC subunits or impaired assembly of OXPHOS complexes shortens RLS [38]. Interestingly, deletion or inhibition of ETC components might also cause increased lifespan. This effect has been associated with reduced ROS production in yeast or activation of stress responses in *C. elegans* and mice (UPR^mt^, the transcription factors HSF-1 or HIF1 pathways) [39–42].

Lon1 protease mediates the degradation of misfolded or damaged proteins in the mitochondrial matrix [43–45]. In agreement with our results, it has been shown that deletion of the budding yeast homolog Pim1 results in accelerated aging [46] whereas LON protease overexpression leads to increased healthspan in the fungus *P. anserina* [47]. In fission yeast, one of the Lon1 substrates identified is Ppr10 which functions as mitochondrial translation activator [48]. We speculate that the low levels of some OXPHOS components observed in *lon1*Δ mutant might be caused by the loss of Ppr10 degradation and in turn defective control of mitochondrial translation. Additionally, the function of Lon protease has been linked with mtDNA stability and transcription, which could explain the reduced content of certain OXPHOS proteins in this mutant [49]. In budding yeast, the absence of OXPHOS subunits in *yta12*Δ mutant has been connected with impaired maturation of its substrate MrpL32, a mitochondrial ribosome subunit [50]. Despite the similar deficiency in OXPHOS components, *yta12*Δ cells showed higher levels of H_2_O_2_ compared to *lon1*Δ mutant (Additional file 5: Fig. S2). This finding is consistent with the different gene expression signatures reported for these two mutants, with *yta12*Δ cells exhibiting a potent induction of the genes involved in the stress response [51].

Our CLS screening has also revealed two long-lived mutants *mgr3*Δ and *yme1*Δ with enhanced oxygen consumption specially in high glucose conditions (Fig. 4B). Multiple studies support a link between increased respiration and lifespan extension [5, 52, 53]. A higher respiration rate has also been detected in long-lived strains of wild yeast isolates [54]. This effect is often a consequence of the activation of stress responses which promote defense mechanisms and/or mitochondrial biogenesis. Similarly, in budding yeast, increased CLS in cells lacking the sirtuin Sir2 has been connected to high respiratory capacity and resistance to different stressors [36, 55–58]. Lifespan extension in *mgr3*Δ and *yme1*Δ mutants seems to be independent of altered ROS levels at the matrix (Additional file 5: Fig. S2), but further work is required to exclude a contribution of stress pathways in these mutants. An additional explanation for the long-lived phenotype of *mgr3*Δ and *yme1*Δ cells could be a higher content of trehalose, an essential storage carbohydrate which has been linked with increased survival during the stationary phase [36].

Another aspect of mitochondrial homeostasis to consider is the degradation of damaged mitochondria by mitophagy. Indeed, impaired mitophagy causes reduced CLS in yeast and accelerated aging in human cells [35, 59]. In contrast, activation of mitophagy might contribute to lifespan extension [33, 34]. For instance, in yeast cells, expression of human Parkin, an E3 ubiquitin ligase required for mitophagy, prolongs CLS under respiratory conditions likely via activation of mitophagy [60]. Our analysis of the protease deletion mutants demonstrates an unambiguous correlation between lifespan and mitophagy: in the short-lived mutants *lon1*Δ and *yta12*Δ, we did not detect signs of mitophagy, while the process was enhanced in the long-lived mutants *mgr3*Δ and *yme1*Δ (Fig. 7A, Additional file 9: Fig. S6A)*.* Moreover, mitophagy inhibition caused by *atg43-1* mutant restored wild-type lifespan in both long-lived mutants, supporting the notion that mitophagy is essential for their long lifespan phenotype (Fig. 7B). In budding yeast, Yme1 has also been linked to mitophagy regulation, but its deletion has the opposite phenotype; this protease is required for the processing of the receptor protein Atg32 and therefore essential for mitophagy [61].

During the characterization of the mitochondrial PQC mutants, we could not prove a direct link between longevity and mitochondrial morphology (Fig. 5E). However, we have identified Yta12 as a regulator of mitochondrial fusion, and future studies will reveal the molecular events driven by this protease regarding mitochondrial morphology. Based on the analysis of respiration and mitophagy, we can conclude that these processes are intimately interconnected (Figs. 4 and 6). Short-lived mutants *lon1*Δ and *yta12*Δ were defective in respiration and mitophagy whereas both activities were stimulated in the long-lived mutants *mgr3*Δ and *yme1*Δ*.* In order to extend lifespan, a high respiratory activity should be accompanied by efficient elimination of damaged mitochondria and the decline of both activities results in severe lifespan shortening. The signaling cascades connecting these two processes, enhanced respiratory activity and mitophagy, are still to be determined.

## Conclusions

In this study, we developed a genome-wide knockout screen searching for fission yeast mutants with altered longevity. Thanks to this approach and the following analysis, we identified four deletion mutants acting at the mitochondria that are implicated in protein quality control. These four mutants exhibited opposite longevity phenotype; while the lack of the proteases Lon1 or Yta12 shortened lifespan, the absence of the protease Yme1 or its adaptor protein Mgr3 caused increased lifespan. Additionally, we demonstrate that mitophagy is required for lifespan extension in wild-type cells upon glucose depletion and in the long-lived mutants *mgr3*Δ and *yme1*Δ under glucose-rich conditions. Hence, this work provides important insights into the modulation of cell longevity by respiration and mitophagy.

## Methods

### Yeast strains

Yeast strains were grown in rich medium (YE) or minimal medium (MM) with the indicated glucose concentrations at 30 °C as previously described [62]. Genotypes of strains used are described in Additional file 11: Table S4.

### Strains and growth conditions for chronological lifespan screening

To perform the chronological aging screening, we used the deletion mutant library of Bioneer version 2.0 which contains 3004 strains. First, the frozen stock of the deletion collection strains was plated into YE plates and incubated at 30 °C. After 2 days, cells were inoculated in 96-well plates containing 150 µl of YE with 3% glucose media. The following day, 10 µl of these cells was transferred into 500 µl of YE with 3% glucose media in 1.3-ml 96-well plates that were incubated at 30 °C with constant agitation. After an overnight incubation (considered logarithmic growth), we started measuring cell viability taking samples every 2 days (days 2, 4, 6, and 8) of stationary growth. Cell viability was measured using propidium iodide (PI) [24]. A total of 10 µl of cultured cells was incubated in 200 µl of PBS with 2 µM of PI during 30 min at 30 °C in the dark. Ten thousand cells were analyzed by flow cytometry, using BD FACSCanto™, and PI staining was monitored using PE-A, which detects red fluorescence. We extracted the data of percentage of living cells to build survival curves, considering that the Log sample is 100% of viable cells. Strains that presented no growth or regrowth after several days in the stationary phase were not included in the analysis. 666 (WT), *sty1*Δ, and *pka1*Δ were included as average, short-lived, and long-lived control strains, respectively. Control strains were included in each of the 96-well plates analyzed.

### Statistical analysis of chronological lifespan screening

The area under the survival curve was calculated as in [63], and it was selected as the parameter to compare the longevity among the strains. Three longevity distributions were defined based on the control strains: average, short-, and long-lived survival. Employing a Bayesian inference model, an equal prior probability for each mutant to belong to each distribution was first established. These priors were then reallocated based on deletion strain survival results, using a Student’s *t*-test. The resulting posterior probabilities were then used to assign each mutant to the model with the highest probability.

### GO enrichment analysis

GO enrichment in Cellular Component and Biological Process was obtained using Metascape [64] from a single list that contains the short-lived (163) and long-lived (309) strains. The parameters used were 3 (minimum overlap), 0.05 (*P*-value cutoff), and 1.5 (minimum enrichment).

### Spot assay

Cells were grown in YE or MM at 30 °C until the stationary phase was reached. The same concentration of cells (OD_600_ 2.5) and 1/10 serial dilutions of culture samples corresponding to logarithmic growth (OD_600_ 0.5) and different days of stationary phase were spotted onto YE or MM plates with the indicated glucose concentration. The spots were allowed to dry, and the plates were incubated at 30 °C for 2–4 days.

### In vivo measurement of matrix H_2_O_2_ levels using MTS-HyPer7

A detailed description of plasmid construction and the experimental procedure has been published [28]. Briefly, plasmid p730 carrying the reporter HyPer7 with the MTS (mitochondrial targeting sequence) of Aco1 was transformed in the mitochondrial protease mutants. HyPer7 has two excitation maxima at 400 and 499 nm and one emission peak at 516 nm [27]. We used excitation filters of 400–10 and 485BP12, combined with an emission filter of EM520 in a FLUOstar OMEGA (BMG Labtech). For the experiments shown here, MM-based early stationary phase pre-cultures were diluted in filtered MM to reach an OD_600_ of 1 after 4–5 duplications. The fluorescence of 190 µl of these cultures was directly monitored in 96-well plates (Krystal Microplate™ 215,003, Porvair Sciences). The two excitation wavelengths were recorded, and after 4 cycles of approximately 2 min each, 10 µl of the indicated H_2_O_2_ treatments were added to accomplish the final concentrations of H_2_O_2_. In two wells, final concentrations of 50 mM dithiothreitol (DTT) and 1 mM of H_2_O_2_ were added as controls of fully reduced and oxidized reporter, respectively. A formula described in [28] was applied to determine the degree of oxidation of the H_2_O_2_ sensor MTS-HyPer7. For each strain, we grew cultures of the wild-type background 972 and performed the same treatments on 96-well plates; after recording, we subtracted the fluorescence values of the wild-type strain to those of the strain expressing the reporter. Graphics represent the mean of three independent experiments.

### Microscopy and image analysis

Samples from cell cultures in the logarithmic phase and after reaching the stationary phase grown in YE with 3% or 1% glucose were harvested by centrifugation and visualized at room temperature. Images were acquired using a Nikon Eclipse 90i microscope equipped with differential interference contrast optics, a PLAN APO VC 100 × 1.4 oil immersion objective, an ORCA-II-ERG camera (Hamamatsu), excitation and emission filters GFP-4050B, mCherry-C (Semrock), and image acquisition software Metamorph 7.8.13 (Gataca Systems). Processing of all images was performed using Fiji (ImageJ, National Institutes of Health) [65]*.* Mitochondria network morphology analysis was conducted with the algorithm MiNa [32]*.* To compare mitochondria of different strains, we chose the parameter summed branch length mean, which represents the mean of the sum of the lengths of branches for each independent structure. To cover a larger volume of the cell, z-stacks of 9 images with 0.3-µm spacing were acquired, deconvolved, and represented in single images as maximum-intensity projections. At least 50 cells from each strain were analyzed. For Mitotracker staining, cell cultures at OD_600_ 0.5 were incubated with 0.1 μg/ml MitoTracker Red CMXRos (Invitrogen) during 30 min. Cells were washed, centrifuged, and resuspended in YE with 3% glucose. For quantification of ΔΨ, the integrity density was measured after segmentation of bright-field images. The Fiji-based macro for image segmentation was designed by Sébastien Tosi (Institute for Research in Biomedicine, Barcelona). At least 100 cells from each strain were analyzed.

### TCA extracts and immunoblot analysis

Modified trichloroacetic acid (TCA) protein extracts were prepared as previously described [66]. Proteins were separated by SDS-PAGE and detected by immunoblotting with monoclonal anti-GFP (Takara). Anti-Sty1 polyclonal antibody [67] was used in the loading control. Antibodies used to detect mitochondrial encoded proteins Cox1, Cox2, Cox3, and Atp6 were described in [48]. StartBright Blue 700 Fluorescence anti-Mouse secondary antibody (Bio-Rad) was used for quantification of mitophagy induction.

### Mitochondria purification

Mitochondria were purified from protoplasts prepared using the Zymolyase and lysing enzymes from *Trichoderma harzianum* as described in [68]. Briefly, protoplasts were broken by 10 strokes using a glass homogenizer, and mitochondria-enriched fraction was obtained after two centrifugation steps at 800 g and 12000 g. For western blot analysis, proteins were precipitated with 10% TCA as described above.

### Oxygen consumption

Oxygen consumption was performed as described previously [10]. Cells were harvested and 2 × 10^7^ cells resuspended in 1 ml of MM to a final OD_600_ of 1. The measurements were made using a Hansatech Oxygraph (Hansatech), with readings being recorded during 10 min. Each one of the measurements was performed from biological triplicates.

### Quantification and statistical analysis

Quantification of western blots was performed using Image Lab software (Bio-Rad). Unless otherwise stated, all experiments were performed at least three times and representative experiments were shown.

## Supplementary Information

**Additional file 1: Table S1. **Deletions strains analyzed in this study.

**Additional file 2: Table S2.** Short and long-lived mutants identified in this study.

**Additional file 3:  Table S3.** Gene Ontology enrichment analysis.

**Additional file 4: Fig. S1.** Deletion of the protease Yta12 results in decreased mitochondrial membrane potential. Quantification of the mitochondrial membrane potential (ΔΨ) of 972 (WT), *lon1*Δ, *yta12*Δ, *mgr3*Δ and *yme1*Δ cells stained with Mitotracker Red. Violin plot represents the integrated density from at least 100 cells of each strain. Significant differences between deletion strains and wild type were determined by two-sided *t*-test (* *p*< 0.05, ** *p*< 0.01, *** *p*<0.001). Right panel shows an example of the segmentation of bright-field (BF) images performed using a Fiji-based macro.

**Additional file 5: Fig. S2.** Analysis of ROS levels in mitochondrial protease mutants. A Levels of H_2_O_2_ in the mitochondrial matrix were determined in 972 (WT), *lon1*Δ and *yta12*Δ, *mgr3*Δ and *yme1*Δ strains expressing the reporter MTS-HyPer7. The indicated concentrations of H_2_O_2_ or DTT were directly added to cultures grown in MM and 96-well imaging plates. Fluorescence was monitored at 30°C for the indicated time points. The degree of probe oxidation (amount of probe oxidized per 1) is shown in the Y-axis (OxD); the starting level of probe oxidation (OxD_0_) for wild-type and *yta12*Δ strains is indicated with dashed lines. For each strain, average data from three biological replicates are shown. B Basal level of probe oxidation (OxD_0_) from Figure S2A strains. Each bar represents mean and SEM from four biological replicates. Significant differences between deletion strains and wild type were determined by two-sided *t*-test (* *p*< 0.05, ** *p*< 0.01, *** *p*<0.001).

**Additional file 6: Fig. S3.** Changes in mitochondrial function and morphology during chronological aging. Fluorescence microscopy of wild-type cells expressing the mitochondrial marker Sdh2-GFP and stained with MitoTracker red to measure membrane potential. Cells were grown in rich media containing 3% glucose and analyzed during logarithmic growth (Log) and stationary phase (days 1, 2 and 3). Maximum and minimum levels were adjusted using Fiji software. Scale bar, 5 μm.

**Additional file 7: Fig. S4.** Quantification of mitochondrial lengths during logarithmic growth. Mitochondrial length of 972 (WT), *dnm1*Δ, *fis1*Δ, *msp1*Δ, *lon1*Δ, *yta12*Δ, *mgr3*Δ and *yme1*Δ strains was determined using the parameter “Summed branch lengths mean” of the MiNa software [32] (*n*>50). Significant differences between deletion strains and wild type were determined by two-sided *t*-test (* *p*< 0.05, ** *p*< 0.01, *** *p*<0.001). Fluorescence microscopy images represent an example of the process of binarization and skeletonization done using MiNa software.

**Additional file 8: Fig. S5.** Monitoring mitophagy induction and chronological lifespan in autophagy mutants. A Inhibition of mitophagy reduces cell longevity under low glucose conditions (1% Glu). Serial dilutions of 972 (WT) and *atg43-1* strains growing in media with 1% glucose were spotted after logarithmic growth (Log) and days 3, 5 and 9 of stationary state. B Fluorescence microscopy of ZD307 (WT), *atg5*Δ, *atg20*Δ *atg24*Δ, *atg24*Δ *atg24b*Δ and *atg20*Δ *atg24*Δ *atg24b*Δ strains expressing the mitochondrial marker Sdh2-mCherry. Cells were grown in rich media containing 3% or 1% glucose and analyzed after logarithmic growth (Log) and day 3 of stationary phase. Images represent maximum-intensity projections of deconvolved z stacks (9 planes, 0.3 μm steps). White arrows indicate vacuoles labeled with mCherry signal. Scale bar, 5 μm. C Cells lacking the Atg proteins involved in organelle-autophagy exhibit a reduced lifespan. ZD307 (WT), *atg20*Δ *atg24*Δ, *atg24*Δ *atg24b*Δ and *atg20*Δ *atg24*Δ *atg24b*Δ strains were grown in rich media with 1% glucose. Serial dilutions corresponding to culture samples from logarithmic phase (Log) and days 3, 5 and 7 of stationary phase were spotted onto rich media plates.

**Additional file 9: Fig. S6.** Mitophagy inhibition in the short-lived strains *lon1*Δ and *yta12*Δ. A Quantification of mitophagy induction using Image lab software. Bar plots represent mean and SD of GFP/Sdh2-GFP ratio from three independent experiments. Significant differences between deletion strains and wild type were determined by two-sided *t*-test (* *p*< 0.05, ** *p*< 0.01,*** *p*<0.001). B *atg43-1* mutant does not reduce further the lifespan of the short-lived mutants *lon1*Δ and *yta12*Δ. Lifespan of 972 (WT), *sty1*Δ, *atg43-1*, *lon1*Δ, *lon1*Δ *yta12*Δ, and *yta12*Δ *atg43-1* strains was measured by propidium iodide staining and FACS. Line plot represents the local regression curves for the average survival of each strain (*n*>10) at different time points. Each survival curve also displays a 95% confidence interval band. Bar plot depicts the average area under the curve of each strain, and error bars represent SD. Significant differences between deletion strains and wild type, and *lon1*Δ and *yta12*Δ mutants versus *lon1*Δ *atg43-1* and *yta12*Δ *atg43-1,* respectively, were determined by two-sided *t*-test (* *p*< 0.05, ** *p*< 0.01, *** *p*<0.001).

**Additional file 10.** Western blots.

**Additional file 11: Table S4.** Yeast strains used in this study.

## Data Availability

All images included in the main and supplementary figures are available as Mendeley dataset (https://data.mendeley.com/datasets/98nyhrvfvz/draft?a=49e13fe8-24b8-45bc-a6c8-94da5b70615c). Materials are available from the corresponding author on request.

## Abbreviations

AAA: ATPases associated with diverse cellular activities
ATG: Autophagy-related
CLS: Chronological lifespan
GFP: Green fluorescent protein
Glu: Glucose
GO: Geneontology
ETC: Electron transport chain
i-AAA: Mitochondrial inner membrane ATPases associated with diverse cellular activities
IM: Mitochondrial inner membrane
IMS: Mitochondrial intermembrane space
Log: Logarithmic growth
Lon1: Lon peptidase 1
m-AAA: Mitochondrial matrix ATPases associated with diverse cellular activities
Mgr3: Mitochondrial genome required 3
MM: Minimal medium
mtDNA: Mitochondrial DNA
MTS: Mitochondrial targeting sequence
OD: Optical density
OM: Mitochondrial outer membrane
OXPHOS: Oxidative phosphorylation
PI: Propidium iodide
PQC: Protein quality control
RLS: Replicative lifespan
ROS: Reactive oxygen species
*S. cerevisiae*: *Saccharomyces cerevisiae*
*S. pombe*: *Schizosaccharomyces pombe*
TCA: Trichloroacetic acid
UPRmt: Mitochondrial unfolded protein response
WT: Wild-type
YE: Yeast extract
Yme1: Yeast mitochondrial escape 1
Yta12: Yeast Tat-binding Analog 12
ΔΨ: Mitochondrial membrane potential
