# Supplementary figures and images for: Antagonistic effects of mitochondrial matrix and intermembrane space proteases on yeast aging
Source: BMC Biol. 2022 Jul 12;20:160. doi: 10.1186/s12915-022-01352-w (PMC9277893; doi:10.1186/s12915-022-01352-w)

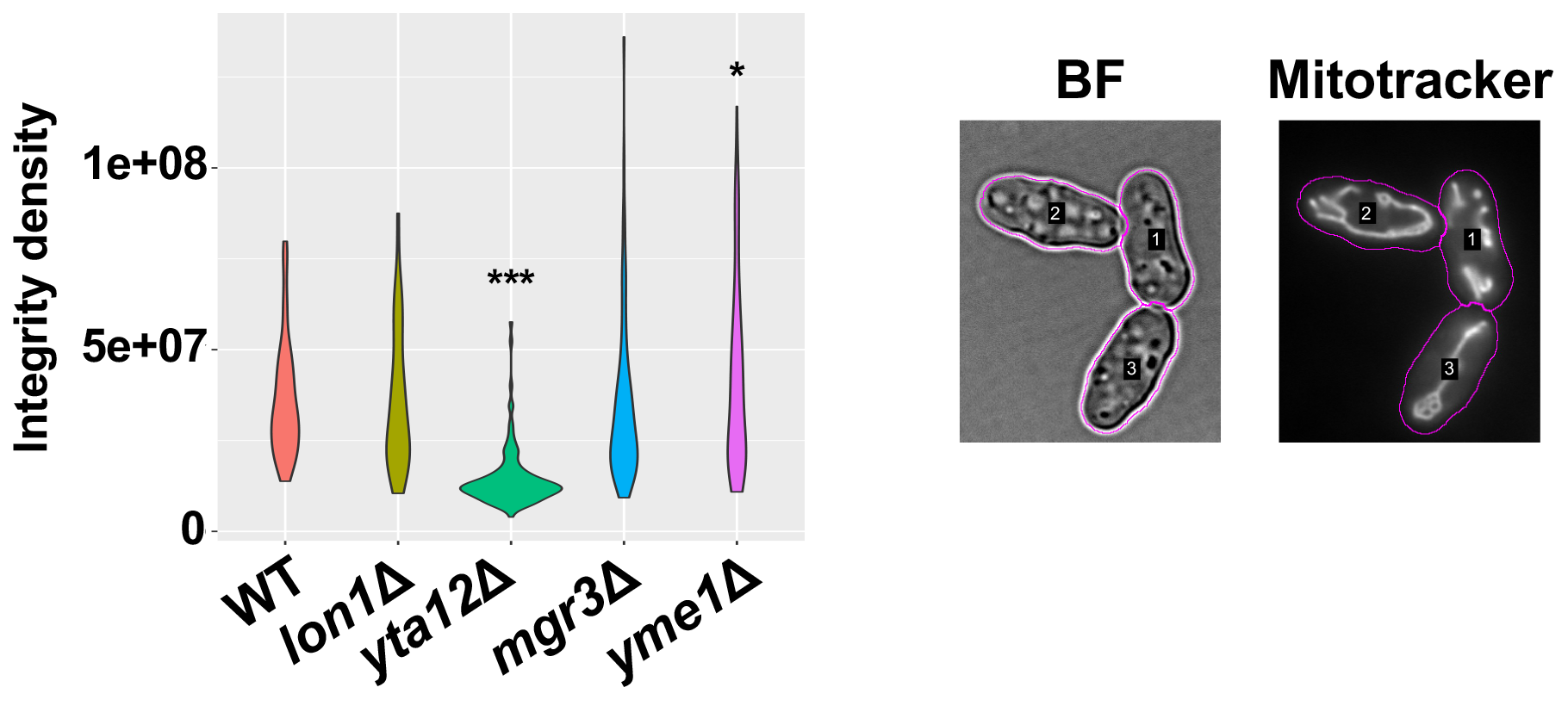

Supplement: Supplementary file 4 — Additional file 4: Fig. S1. Deletion of the protease Yta12 results in decreased mitochondrial membrane potential. Quantification of the mitochondrial membrane potential (ΔΨ) of 972 (WT), lon1Δ, yta12Δ, mgr3Δ and yme1Δ cells stained with Mitotracker Red. Violin plot represents the integrated density from at least 100 cells of each strain. Significant differences between deletion strains and wild type were determined by two-sided t-test (* p< 0.05, ** p< 0.01, *** p<0.001). Right panel shows an example of the segmentation of bright-field (BF) images performed using a Fiji-based macro. [file 12915_2022_1352_MOESM4_ESM.tif]

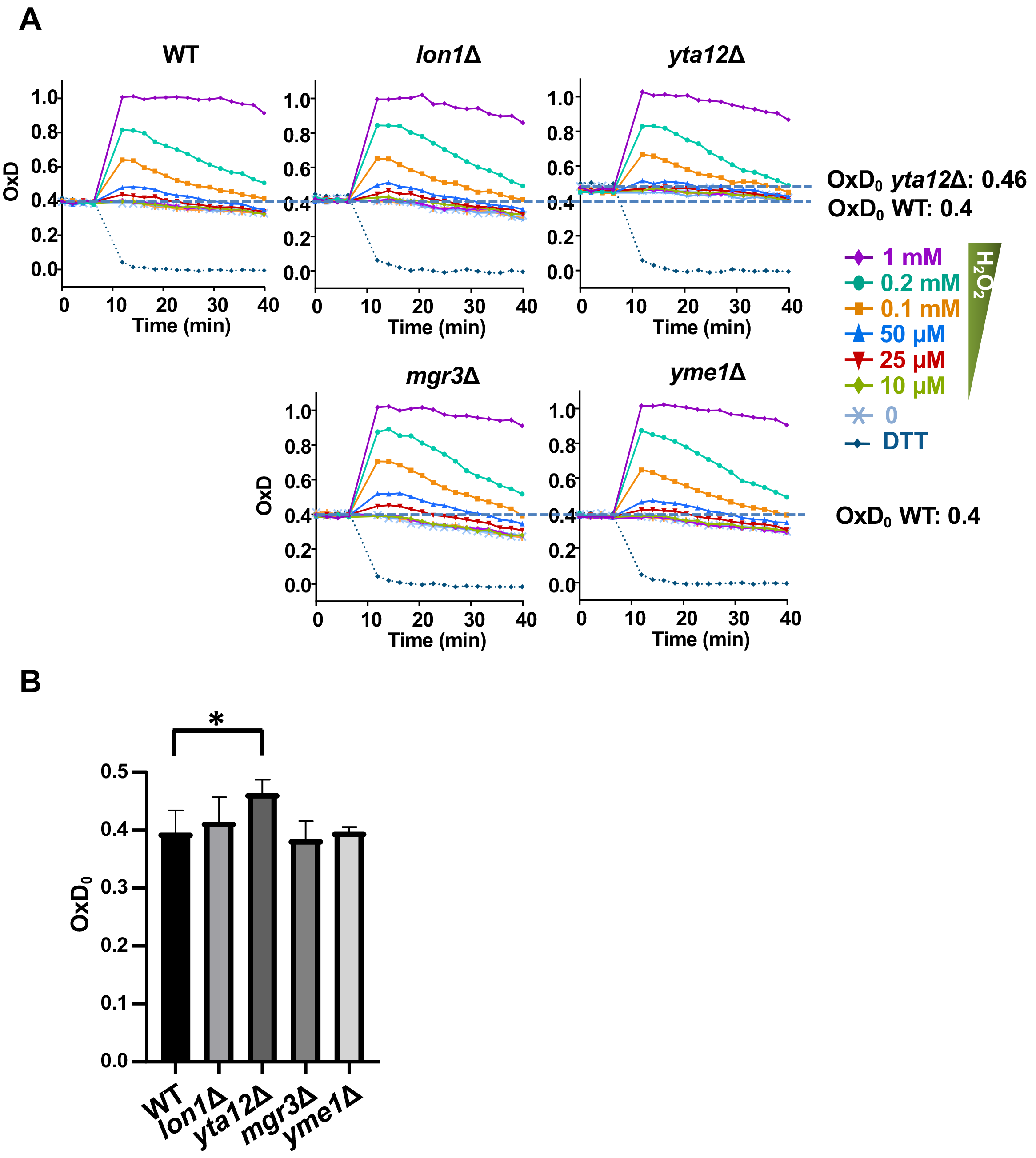

Supplement: Supplementary file 5 — Additional file 5: Fig. S2. Analysis of ROS levels in mitochondrial protease mutants. A Levels of H2O2 in the mitochondrial matrix were determined in 972 (WT), lon1Δ and yta12Δ, mgr3Δ and yme1Δ strains expressing the reporter MTS-HyPer7. The indicated concentrations of H2O2 or DTT were directly added to cultures grown in MM and 96-well imaging plates. Fluorescence was monitored at 30°C for the indicated time points. The degree of probe oxidation (amount of probe oxidized per 1) is shown in the Y-axis (OxD); the starting level of probe oxidation (OxD0) for wild-type and yta12Δ strains is indicated with dashed lines. For each strain, average data from three biological replicates are shown. B Basal level of probe oxidation (OxD0) from Figure S2A strains. Each bar represents mean and SEM from four biological replicates. Significant differences between deletion strains and wild type were determined by two-sided t-test (* p< 0.05, ** p< 0.01, *** p<0.001). [file 12915_2022_1352_MOESM5_ESM.tif]

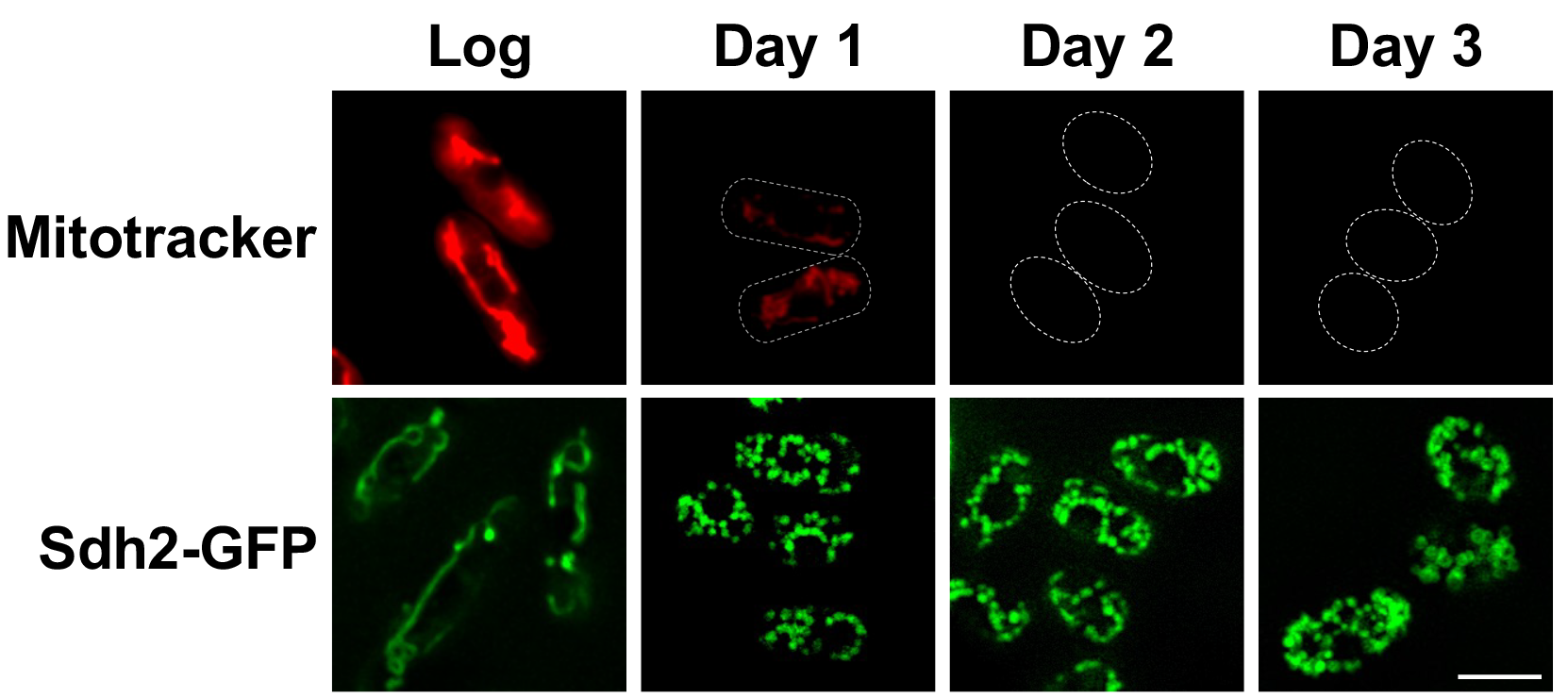

Supplement: Supplementary file 6 — Additional file 6: Fig. S3. Changes in mitochondrial function and morphology during chronological aging. Fluorescence microscopy of wild-type cells expressing the mitochondrial marker Sdh2-GFP and stained with MitoTracker red to measure membrane potential. Cells were grown in rich media containing 3% glucose and analyzed during logarithmic growth (Log) and stationary phase (days 1, 2 and 3). Maximum and minimum levels were adjusted using Fiji software. Scale bar, 5 μm. [file 12915_2022_1352_MOESM6_ESM.tif]

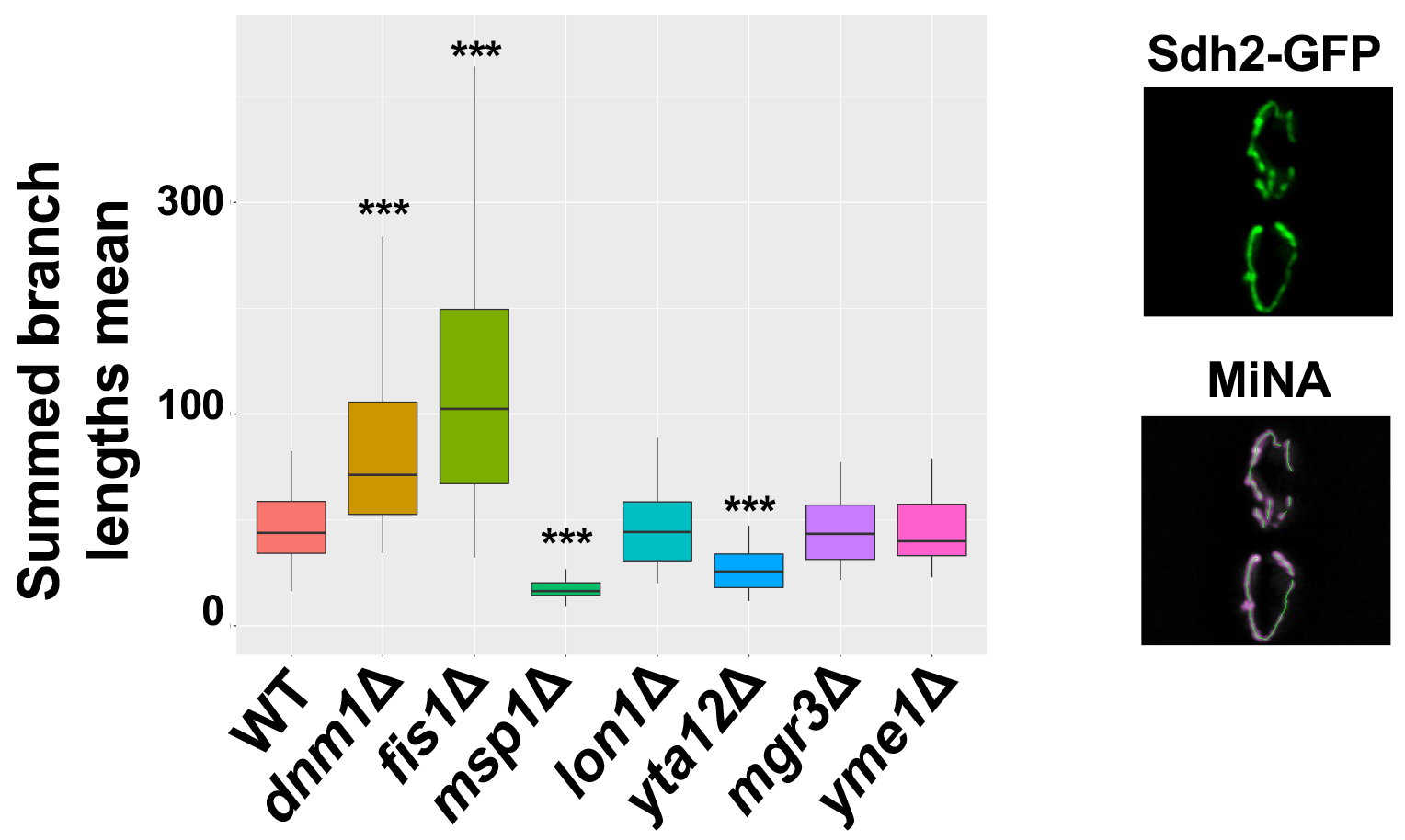

Supplement: Supplementary file 7 — Additional file 7: Fig. S4. Quantification of mitochondrial lengths during logarithmic growth. Mitochondrial length of 972 (WT), dnm1Δ, fis1Δ, msp1Δ, lon1Δ, yta12Δ, mgr3Δ and yme1Δ strains was determined using the parameter “Summed branch lengths mean” of the MiNa software [32] (n>50). Significant differences between deletion strains and wild type were determined by two-sided t-test (* p< 0.05, ** p< 0.01, *** p<0.001). Fluorescence microscopy images represent an example of the process of binarization and skeletonization done using MiNa software. [file 12915_2022_1352_MOESM7_ESM.tif]

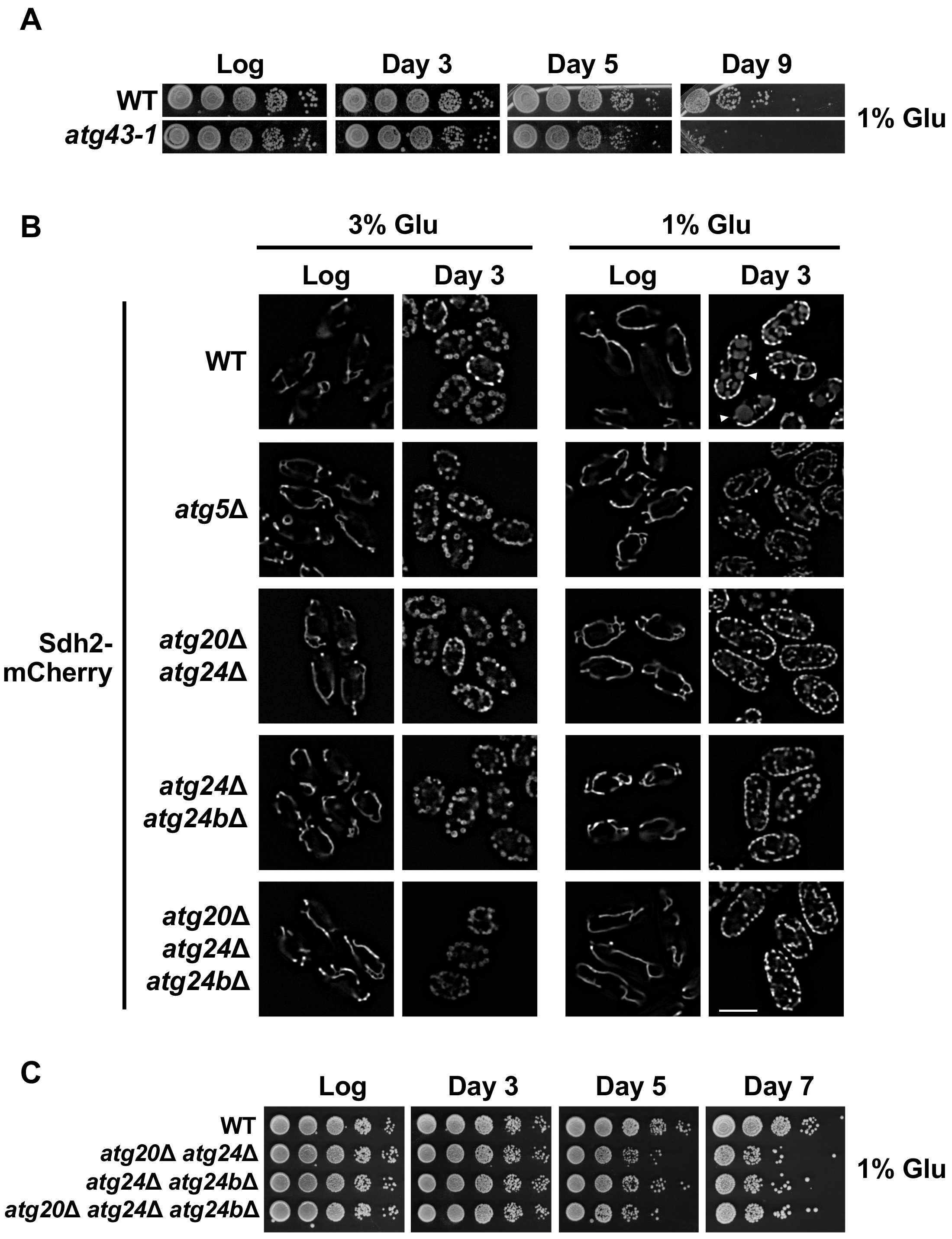

Supplement: Supplementary file 8 — Additional file 8: Fig. S5. Monitoring mitophagy induction and chronological lifespan in autophagy mutants. A Inhibition of mitophagy reduces cell longevity under low glucose conditions (1% Glu). Serial dilutions of 972 (WT) and atg43-1 strains growing in media with 1% glucose were spotted after logarithmic growth (Log) and days 3, 5 and 9 of stationary state. B Fluorescence microscopy of ZD307 (WT), atg5Δ, atg20Δ atg24Δ, atg24Δ atg24bΔ and atg20Δ atg24Δ atg24bΔ strains expressing the mitochondrial marker Sdh2-mCherry. Cells were grown in rich media containing 3% or 1% glucose and analyzed after logarithmic growth (Log) and day 3 of stationary phase. Images represent maximum-intensity projections of deconvolved z stacks (9 planes, 0.3 μm steps). White arrows indicate vacuoles labeled with mCherry signal. Scale bar, 5 μm. C Cells lacking the Atg proteins involved in organelle-autophagy exhibit a reduced lifespan. ZD307 (WT), atg20Δ atg24Δ, atg24Δ atg24bΔ and atg20Δ atg24Δ atg24bΔ strains were grown in rich media with 1% glucose. Serial dilutions corresponding to culture samples from logarithmic phase (Log) and days 3, 5 and 7 of stationary phase were spotted onto rich media plates. [file 12915_2022_1352_MOESM8_ESM.tif]

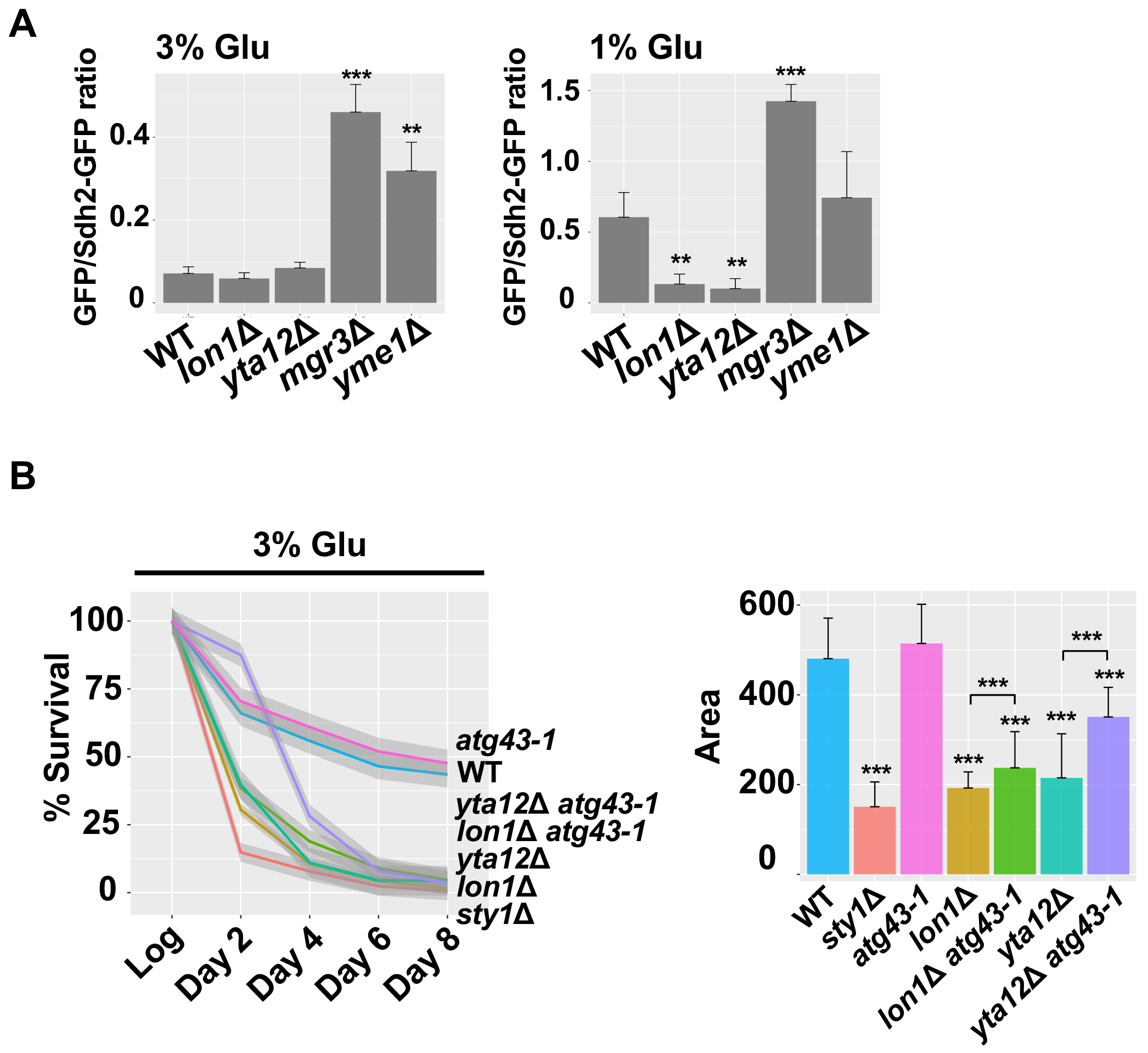

Supplement: Supplementary file 9 — Additional file 9: Fig. S6. Mitophagy inhibition in the short-lived strains lon1Δ and yta12Δ. A Quantification of mitophagy induction using Image lab software. Bar plots represent mean and SD of GFP/Sdh2-GFP ratio from three independent experiments. Significant differences between deletion strains and wild type were determined by two-sided t-test (* p< 0.05, ** p< 0.01,*** p<0.001). B atg43-1 mutant does not reduce further the lifespan of the short-lived mutants lon1Δ and yta12Δ. Lifespan of 972 (WT), sty1Δ, atg43-1, lon1Δ, lon1Δ yta12Δ, and yta12Δ atg43-1 strains was measured by propidium iodide staining and FACS. Line plot represents the local regression curves for the average survival of each strain (n>10) at different time points. Each survival curve also displays a 95% confidence interval band. Bar plot depicts the average area under the curve of each strain, and error bars represent SD. Significant differences between deletion strains and wild type, and lon1Δ and yta12Δ mutants versus lon1Δ atg43-1 and yta12Δ atg43-1, respectively, were determined by two-sided t-test (* p< 0.05, ** p< 0.01, *** p<0.001). [file 12915_2022_1352_MOESM9_ESM.tif]
