# Supplementary material for: Antagonistic effects of mitochondrial matrix and intermembrane space proteases on yeast aging
Source: BMC Biol. 2022 Jul 12;20:160. doi: 10.1186/s12915-022-01352-w (PMC9277893; doi:10.1186/s12915-022-01352-w)

Fig. 4E

α-Cox1

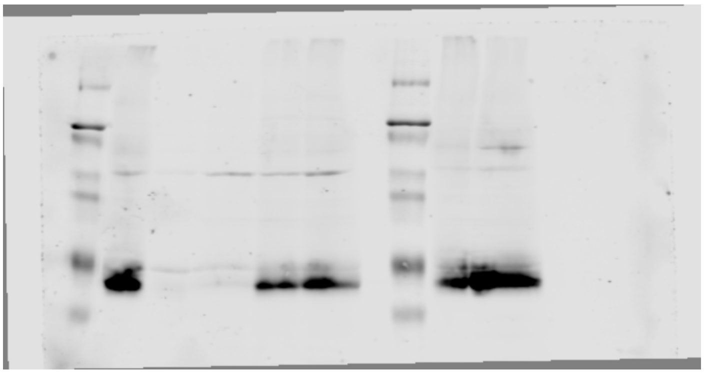

α-Cox2

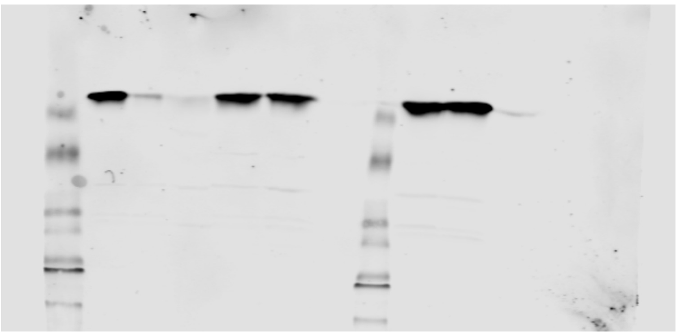

α-Cox3

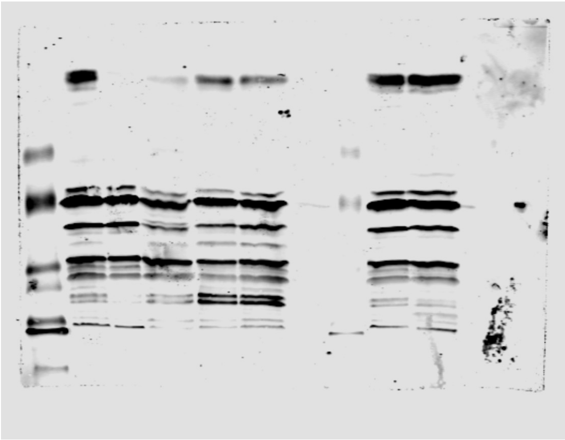

α-Atp6

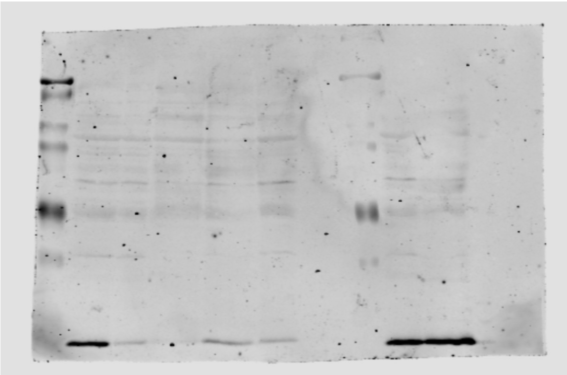

Sdh2-GFP

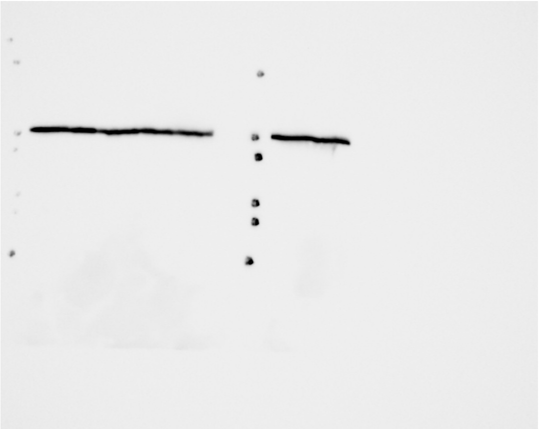

## Fig. 6A

Sdh2-GFP

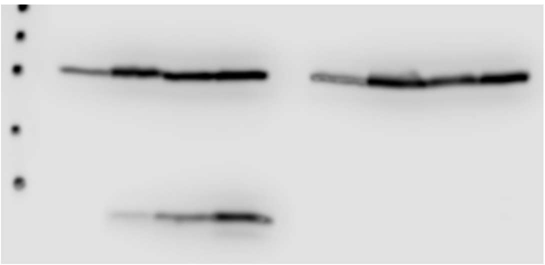

αSty1

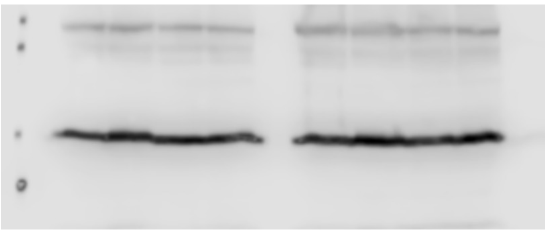

## Fig. 6B

Sdh2-GFP

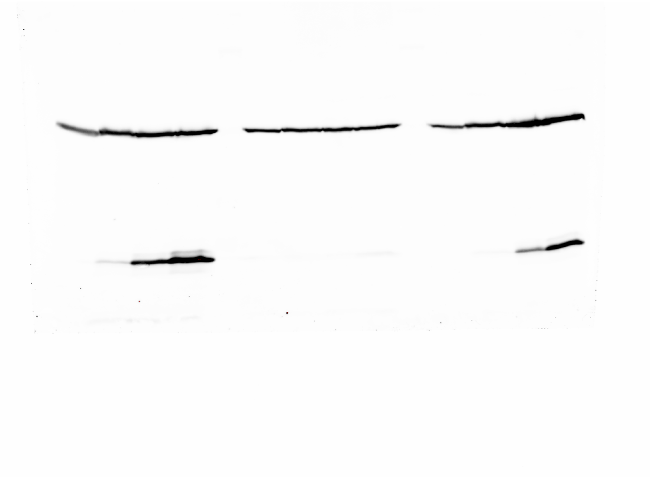

αSty1

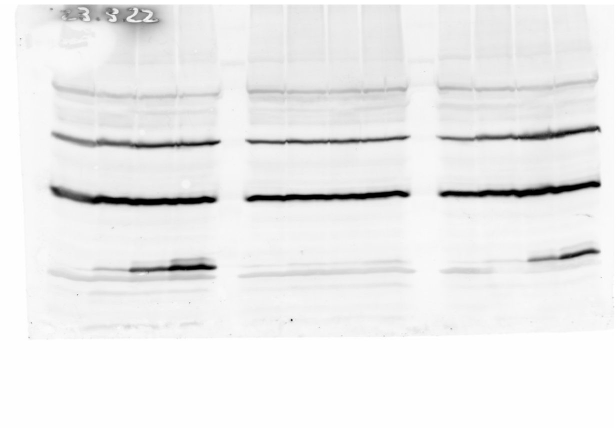

## Fig. 6D

Sdh2-GFP

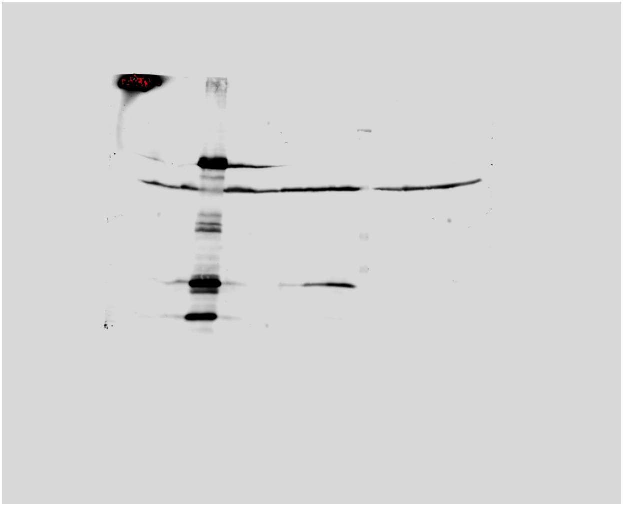

Ponceau

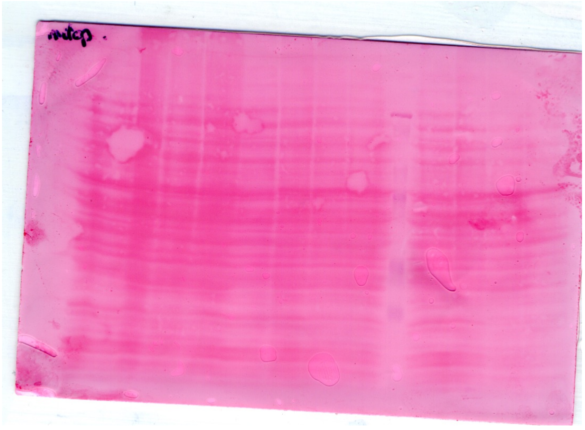

Fig. 7A

Sdh2-GFP

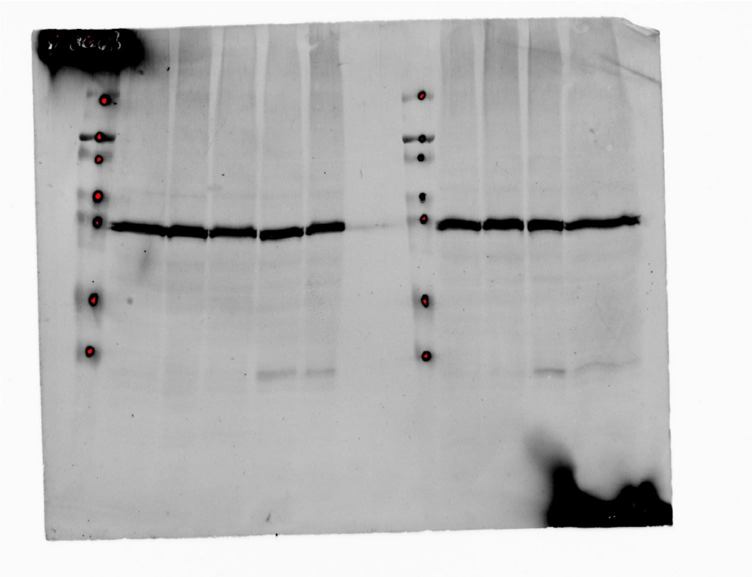

Ponceau

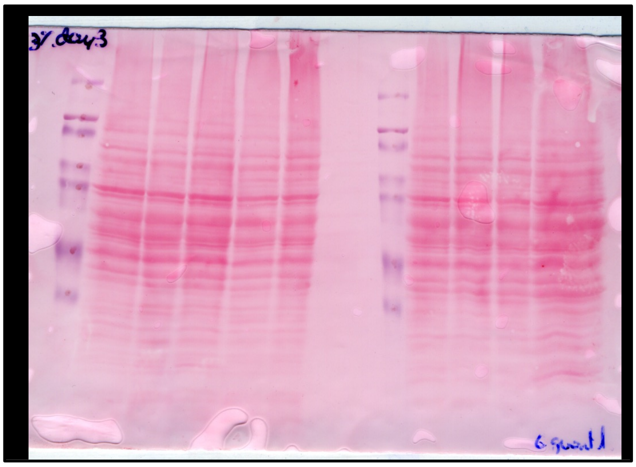

Sdh2-GFP

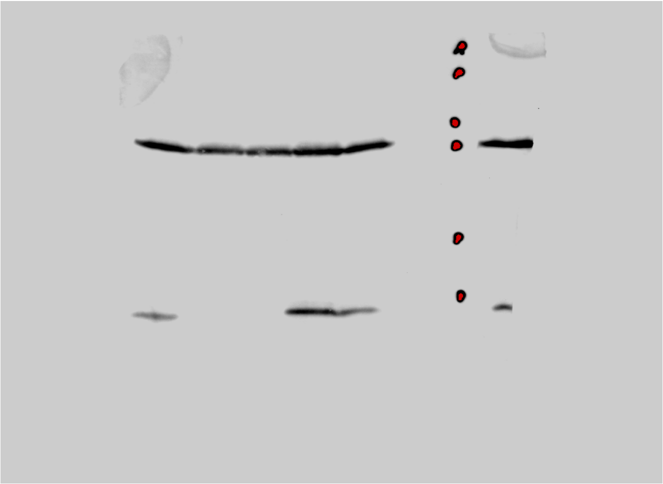

Ponceau

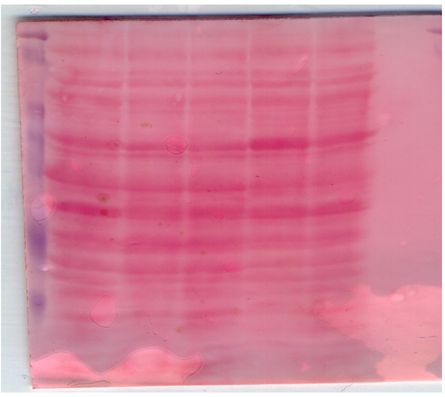

Supplement: Supplementary file 10 — Additional file 10. Western blots. [file 12915_2022_1352_MOESM10_ESM.pdf]
