# Supplementary material for: Antagonistic effects of mitochondrial matrix and intermembrane space proteases on yeast aging
Source: BMC Biol. 2022 Jul 12;20:160. doi: 10.1186/s12915-022-01352-w (PMC9277893; doi:10.1186/s12915-022-01352-w)
Supplement: Supplementary file 11 — Additional file 11: Table S4. Yeast strains used in this study. [file 12915_2022_1352_MOESM11_ESM.docx]

**Additional file 11: Table S4.**

Yeast strains used in this study.

| Strain | Genotype | Origen |
| --- | --- | --- |
| 972 (WT) | *972 h-* | (Leupold, 1970) |
| 666 (WT) | *h+ ade6-M21x ura4-D18 leu1-32* | Bioneer collection |
| *sty1*Δ | *h+ sty1::kanMX6 ade6-M21x ura4-D18 leu1-32* | Bioneer collection |
| *pka1*Δ | *h+ pka1::kanMX6 ade6-M21x ura4-D18 leu1-32* | Bioneer collection |
| AV18 | *h- sty1::kanMX6* | (Zuin et al., 2005) |
| AZ88 | *h+ cox6::kanMX6* | (Zuin et al., 2008) |
| EP48 | *h+ pyp1::natMX6* | (Boronat et al., 2020) |
| MCS17 | *h+ lon1::kanMX6* | This work |
| MCS19 | *h+ mgr3::kanMX6* | This work |
| MCS46 | *h- yme1::natMX6* | This work |
| MCS47 | *h- sdh2-GFP::natMX6* | This work |
| MCS52 | *h- yta12::natMX6* | This work |
| MV19 | *h? sdh2-GFP::natMX6 cpy1-mCherry::kanMX6* | This work |
| MV23 | *h+ lon1::kanMX6 sdh2GFP::natMX6* | This work |
| MV24 | *h- mgr3::kanMX6 sdh2GFP::natMX6* | This work |
| MV38 | *h? yme1::natMX6 sdh2GFP::kanMX6* | This work |
| MV39 | *h^-^ yta12::natMX6 sdh2GFP::kanMX6* | This work |
| MV40 | *h^-^ dnm1::natMX6 sdh2GFP::kanMX6* | This work |
| MV47 | *h+ ∆atg8::kanMX6 sdh2GFP::natMX6* | This work |
| MV67 | *h^-^ fis1::kanMX6 sdh2-GFP::natMX6* | This work |
| MV70 | *h^-^ msp1::kanMX6 sdh2-GFP::natMX6* | This work |
| MV71 | *h+ fis1::kanMX6* | This work |
| MV72 | *h- dnm1::natMX6* | This work |
| MV73 | *h? msp1::kanMX6* | This work |
| MV75 | *h+ atg43-1::kanMX6* | This work |
| MV78 | *h? atg43-1::kanMX6 mgr3::natMX6* | This work |
| MV79 | *h? atg43-1::kanMX6 yme1::natMX6* | This work |
| MV92 | *h? atg43-1::kanMX6 yta12::natMX6* | This work |
| MV94 | *h? atg43-1::kanMX6 lon1::hphMX6* | This work |
| ZD196 | *h+  atg24::natMX6 atg24b::hphMX6 Sdh2-mCherry::kanMX6 his3-D1 leu1-32* | (Zhao et al., 2016) |
| ZD197 | *h+ atg20::kanMX6 atg24::natMX6 atg24b::hphMX6 sdh2-mCherry::kanMX6 his3-D1 leu1-32* | (Zhao et al., 2016) |
| ZD199 | *h? atg24::hphMX6 atg20::kanMX6 sdh2-mCherry::kanMX6 his3-D1 leu1-32* | (Zhao et al., 2016) |
| ZD306 | *h? atg5::hphMX6 sdh2-mCherry::kanMX6 cpy1-venus::natMX6 CFP-atg8::leu+ his3-D1 leu1-32* | (Zhao et al., 2016) |
| ZD307 | *h- sdh2-mCherry::kanMX6 cpy1-venus::natMX6 CFP-atg8::leu+ his3-D1 leu1-32* | (Zhao et al., 2016) |

Leupold U. Genetical Methods for Schizosaccharomyces pombe. Methods Cell Biol. 1970;4:169–77.

Zuin A, Vivancos AP, Sansó M, Takatsume Y, Ayté J, Inoue Y, et al. The Glycolytic Metabolite Methylglyoxal Activates Pap1 and Sty1 Stress Responses in Schizosaccharomyces pombe. J Biol Chem. 2005;280:36708–13.

Zuin A, Gabrielli N, Calvo IA, García-Santamarina S, Hoe K-L, Kim DU, et al. Mitochondrial Dysfunction Increases Oxidative Stress and Decreases Chronological Life Span in Fission Yeast. Plos One. 2008;3:e2842.

Boronat S, Marte L, Vega M, García-Santamarina S, Cabrera M, Ayté J, et al. The Hsp40 Mas5 Connects Protein Quality Control and the General Stress Response through the Thermo-sensitive Pyp1. Iscience. 2020;23:101725.

Zhao D, Liu X-M, Yu Z-Q, Sun L-L, Xiong X, Dong M-Q, et al. Atg20- and Atg24-family proteins promote organelle autophagy in fission yeast. J Cell Sci. 2016;129:4289–304.
